# From Chemistry to Functionality: HPLC–DAD/LC–MS/MS Characterization of Bee Product-Enriched *Prunus spinosa* L. Kombucha with In Vitro Antidiabetic Activity and Bioaccessibility

**DOI:** 10.1021/acsomega.6c00008

**Published:** 2026-02-18

**Authors:** Melikenur Türkol, Çiğdem Yıldırım Maviş, Seydi Yıkmış

**Affiliations:** † Nutrition and Dietetics, Faculty of Health Sciences, Halic University, Istanbul 34060, Türkiye; ‡ Department of Food Technology, Tekirdag Namik Kemal University, Tekirdag 59830, Türkiye

## Abstract

This study aimed to sensorially optimize kombucha tea enriched with *Prunus spinosa* L. (blackthorn) juice and bee products (propolis, pollen, and bee bread), and to elucidate the product’s bioactive profile and functional potential (antidiabetic activity and in vitro bioaccessibility) using advanced analytical approaches. The formulations were optimized using response surface methodology (RSM). Alongside the evaluation of total phenolic content (TPC), total flavonoid content (TFC), DPPH• radical scavenging activity, and total antioxidant capacity (TAC), detailed compositional analyses were conducted. The phenolic composition was quantitatively characterized by HPLC–DAD, organic acid profiles were determined using HPLC, and free amino acids were quantified by triple quadrupole LC–MS/MS. In sensory evaluation, the bee bread-enriched kombucha (BB-BKT) received the highest general acceptability score (8.32), with a taste score of 8.25. At the same time, the pollen-enriched sample (PL-BKT) stood out for its smell (8.21). Regarding bioactive content, BB-BKT exhibited the highest values, with TPC of 257.10 mg GAE/100 mL, TFC of 36.16 mg CE/100 mL, and DPPH• activity of 57.05 μmol TE/mL. According to HPLC–DAD results, total phenolics increased from 106.35 μg/mL in the control sample to 159.23–186.15 μg/mL after enrichment. Chlorogenic acid (79.04 μg/mL) was prominent in BB-BKT, catechin hydrate (121.2 μg/mL) in PL-BKT, and quercetin (47.32 μg/mL) and naringenin (17.59 μg/mL) in the propolis-enriched sample (PB-BKT). LC–MS/MS analysis revealed that PL-BKT was the richest sample in free amino acids, with significant increases in proline (47.37 mg/100 mL), leucine (4.10 mg/100 mL), and lysine (4.54 mg/100 mL). In antidiabetic activity assays, the propolis-enriched sample (PB-BKT) showed the highest activity, with 40.7% inhibition of α-glucosidase and 42.02% inhibition of α-amylase. Although a gradual decrease in bioactives was observed during in vitro digestion, the preservation of phenolic and flavonoid recovery generally within the ∼21–30% range suggests that optimized enrichment may support postdigestive functional effects.

## Graphical abstract

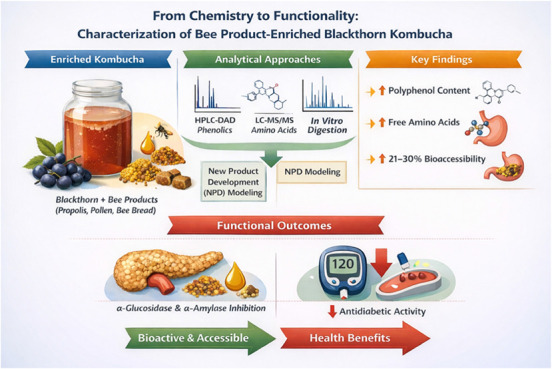

## 1 Introduction

Kombucha tea, a traditionally fermented beverage, is believed to have originated as early as 200–300 BC. In recent decades, it has attracted growing global interest due to its recognized health-promoting properties and its deep cultural roots.^1,2^ Sweetened tea derived from *Camellia sinensis* is transformed into kombucha through fermentation carried out by a SCOBY (Symbiotic Culture of Bacteria and Yeast), a mixed culture of yeasts and bacteria that forms a cellulose-rich biofilm throughout the process.^3^ SCOBY represents a symbiotic consortium of microorganisms that comprises various yeast genera, including *Saccharomyces*, *Schizosaccharomyces*, *Zygosaccharomyces*, *Brettanomyces/Dekkera*, and *Pichia*, as well as both acetic and lactic acid bacteria.^2^ During fermentation, the sugars formed contribute a mild sweetness to the tea, whereas the organic acids synthesized by acetic acid bacteria are responsible for its distinctive sour flavor.^4^ Kombucha tea is traditionally brewed using black tea; however, alternative versions can also be produced with other types of tea.^5^ It has been reported that herbal infusions and fruit juices can also be used for fermentation with the kombucha culture, and due to their chemical composition, these substrates have been shown to serve as promising alternatives.^6^ The antioxidant potential of kombucha tea is primarily linked to its abundant phenolics, with flavonoids playing a significant contributory role.^7^ A high anthocyanin content tends to enhance the antioxidant capacity.^8^ The antioxidant activity of kombucha tea can be improved by incorporating anthocyanin-rich fruits. Accordingly, the use of wild fruits with high anthocyanin content—such as *Prunus spinosa* L. (blackthorn), *Prunus laurocerasus* (cherry laurel), and *Rubus idaeus* (red raspberry)—has been considered a practical way to improve the beverage’s nutritional, functional and sensorial properties.^9^

Fruits are essential for preserving global biodiversity and are a fundamental element supporting the functioning and continuity of natural ecosystems. Due to their rich nutrient composition, distinctive flavors and aromas, medicinal potential, and health-promoting effects, wild edible fruits are regarded by consumers as valuable and functional food sources.^10–12^ Türkiye is one of the most significant centers of genetic diversity for wild plum species, including *Prunus cerasifera* and *Prunus spinosa* L. Within this group, *P. spinosa* L., commonly known as blackthorn, is a drought-tolerant ecotype producing small, sour fruits. In addition to being eaten fresh, the fruit and other parts of the plant are used to make marmalade, jelly, fruit juice, preserves, and pastries. Furthermore, blackthorn flowers, harvested in spring and dried, can be used to make tea. This species is notably rich in chemical compounds.^13^ Fermentation is widely recognized as an effective process for improving food quality, as it can promote the development of novel probiotic microorganisms and enhance the nutritional value of foods, thereby contributing positively to human health. Previous studies have demonstrated that the nutritional quality of various vegetables is significantly improved following fermentation.^14,15^ Among food components, proteins are particularly susceptible to fermentation, during which they are converted into more desirable bioactive peptides with enhanced biological activity. In addition, fermentation has been shown to reduce the content of antinutritional factors, such as tannins, phytates, and certain proteins, thereby improving overall nutritional quality.^15,16^ Fermentation represents the most appropriate method for improving pollen quality, as it naturally takes place in the hive following pollen collection by bees. This process converts bee-collected pollen into bee bread and despite their common botanical origin, fermentation primarily accounts for the differences observed in their amino acid, fatty acid, mineral, and phenolic profiles.^17,18^

It is reported that the total phenolics and anthocyanin content of *P. spinosa* L. fruit, which contributes significantly to its antioxidant capacity, is based on its rich content of cyanidin-3-glucoside (11.4%), cyanidin-3-rutinoside (53.5%), and peonidin-3-rutinoside (32.4%).^19^ Previous research has demonstrated that fruits of *P. spinosa* L. contain high levels of anthocyanins, flavonols, flavones, phenolic acids, and other flavonoid compounds.^13,20^

Increasing consumer interest in natural products has driven a substantial rise in research focused on the nutritional composition and health-related benefits of bee-derived materials. Natural resources such as plants, microorganisms, bee products, and algae constitute essential sources of a variety of bioactive compounds with diverse structural properties and biological functions.^21^ Bee products are an important natural product group known for their nutritional and medicinal properties. *Apis mellifera* L. (Honeybees) produce a variety of byproducts, including bee pollen, bee bread, royal jelly, propolis, and beeswax, in addition to honey. The therapeutic properties of these products have been recognized by civilizations thousands of years ago and are widely used today as dietary supplements and health support products.^22^ These constituents exhibit a broad spectrum of biological activities, such as antimicrobial, anti-inflammatory, antitumor, and antioxidant effects.^23^ Throughout history, it has served multiple roles in apitherapy and has been highly valued for its therapeutic, cosmetic, and nutritional benefits.^24,25^ Recent reviews have reported that honey bee products, including propolis, bee pollen, and royal jelly, exhibit anti-inflammatory, immunomodulatory, and cardiometabolic benefits in preclinical and clinical studies.^26,27^

Propolis is a naturally occurring material that honeybees gather from the resins of different trees and the buds of various plants.^28,29^ Propolis is a chemically complex matrix comprising more than 300 identified components, including flavonoids, phenolic acids, and their ester derivatives, and is associated with a wide range of biological activities such as antiviral, antioxidant, antimicrobial, anti-inflammatory, antitumor, hepatoprotective, and immunomodulatory effects.^30–34^ Minerals and vitamins are also present in high concentrations in propolis.^28^ Bee pollen is formed when bees agglutinate pollen grains using their saliva, nectar, or honey and store them in granular form in their honeycomb cells. The key role of bee pollen is to ensure the colony’s survival by providing the necessary nutrients, thereby supporting development and maintenance processes.^35^ Bee pollen is a natural product rich in bioactive compounds. More than 250 bioactive compounds have been identified in bee pollen derived from different plant species. These basic chemical components include lipids, amino acids, proteins, carbohydrates, fatty acids, phenolic compounds, carotenoids, flavonoids, vitamins, and bioelements.^36^ Exhibits antihyperglycemic, anti-inflammatory, hepatoprotective, nephroprotective, and anticancer properties thanks to its rich and versatile component mixture.^37,38^ Many of these have demonstrated antioxidant, antimicrobial, antidiabetic. They have shown promising results in the treatment of diabetes and obesity, and their potential as natural therapeutic agents has been evaluated. They have been found to increase insulin sensitivity, reduce oxidative stress, regulate appetite, adjust obesity-related hormone levels, and strengthen antioxidant defense systems.^39,40^ The antimicrobial effect of bee bread has been extensively studied. Bakour et al. found that different bacterial species and fungi are sensitive to the hydromethanolic bee bread extract.^41^ A vital bee byproduct, bee bread is formed after the pollen gathered by bees is enriched with nectar and bee saliva, leading to an ensuing lactic acid fermentation process inside the hives.^42^ Bee bread is high in nutrition, with significant carbohydrates, proteins and lipids. It is a rich source of vitamins, phenolic compounds, minerals and essential amino acids, and exhibits multiple chemical activities.^43,44^

The Response Surface Method (RSM) is one of the statistical optimization approaches. This method uses empirical modeling techniques to identify relationships between independent variables and the system response using experimental data. It also allows for determining optimal conditions for achieving the desired outcome by evaluating the effects of process variables on the system response.^45–47^

Previous studies have not extensively investigated the sensory parameters (color, taste, smell, and general acceptance) of kombucha tea enriched with *P. spinosa* L. and bee products using RSM. The primary aim of this study was to optimize the sensory properties of kombucha formulations enriched with *P. spinosa* L. and bee-derived ingredients (bee bread, propolis, and pollen) using RSM to determine the most acceptable formulation. While many studies have examined kombucha enriched with various fruit and plant infusions, none have specifically investigated the impact of *P. spinosa* L. and bee products on kombucha’s sensory and bioactive properties. The study also evaluated the total phenolic content (TPC), total flavonoid content (TFC), antioxidant activity (DPPH•), total anthocyanin content (TAC), phenolic profile (HPLC), microbiological quality, and levels of organic acids amino acids and in vitro bioaccessibility in the developed samples, as well as their antidiabetic activity. RSM-based optimization offers a new methodological approach. It is expected to yield valuable data. This data will improve the functional and disease-fighting properties of kombucha.

## 2 Materials and Methods

### 2.1 Preparation of *P. spinosa* L. Juice

*P. spinosa* L. fruits were harvested from Tekirdağ, Türkiye, during their ripening season. Fresh fruits were homogenized for 30 s at low speed using a laboratory blender (ISO LAB Blender, 602.21.001) to obtain a fruit pulp. The remaining pulp was diluted with sterile distilled water at a 1:1 ratio and subsequently filtered. The obtained concentrate was transferred into sterile 50 mL screw-capped sample containers and stored at −18 °C until further analysis. All analyses were performed in the laboratories of the Department of Nutrition and Dietetics, Tekirdağ Namık Kemal University.

### 2.2 Preparation of Kombucha Tea Enriched with *P. spinosa* L

The SCOBY is a cellulose-based biofilm containing a consortium of symbiotic bacteria and yeasts. It is produced as a byproduct of the kombucha fermentation process.^48^ Kombucha tea enriched with *P. spinosa* L. was prepared by adapting the traditional kombucha production process with some modifications based on previous studies. *Camellia sinensis* (tea leaves) were infused in hot water under controlled temperature and time conditions. While the infusion was still hot, sucrose was added and allowed to dissolve completely. The inoculum and the kombucha culture (SCOBY) were added after cooling to room temperature. The fermentation vessel was covered with an air-permeable material and incubated at 25–30 °C for 10–14 days.^9,49^ The process began by dissolving 60 g of sucrose in 1 l of hot drinking water (maintained at 98 °C), followed by a 15 min pasteurization period. Subsequently, 10 g of *Camellia sinensis* (black tea) was infused in this solution at 95 °C for 12 min and then filtered. After cooling to room temperature, the black tea infusion was inoculated with 100 mL of kombucha tea (previously fermented liquid) and the kombucha culture. The kombucha culture was obtained from a previous black tea fermentation that lasted 14 days. The tea base was then enriched with 10% (w/w) *P. spinosa* L. juice concentrate and inoculated with 10% (v/v) kombucha culture. Fermentation was conducted at 28 ± 2 °C under static conditions. The *P. spinosa* L.-enriched kombucha tea was used as the experimental sample, and plain kombucha served as the control. Kombucha enriched with *P. spinosa* L. was used as the control in order to specifically evaluate the additional functional effects of bee products beyond the phytochemical contribution of the fruit.

### 2.3 Experimental Design (Optimization of *P. spinosa* L. and Bee Product-Enriched Kombucha Tea Using RSM)

Bee products, including propolis, pollen, and bee bread, were commercially obtained from local suppliers. The kombucha tea enriched with *P. spinosa* L. was used as the control sample. RSM implemented in Minitab Statistical Analysis Software (version 18.1.1) was employed to examine the formulation of kombucha teas enriched with propolis, pollen, and bee bread, with the aim of assessing their effects on sensory characteristics. The amounts of *P. spinosa* L. juice, propolis, pollen, and bee bread concentrations were designated as independent variables. At the same time, the sensory parameters (color, taste, smell, and general acceptance) were considered as dependent variables. A Central Composite Design (CCD) was adopted to optimize the production of blackthorn-enriched kombucha tea. The experimental framework consisted of a two-factor design with five levels, encompassing a total of 13 experimental runs. Blackthorn-enriched kombucha samples were prepared using *P. spinosa* L. concentrations of 8%, 10%, 12%, 14%, and 16% (w/w) and tea concentrations of 8, 9.5, 11, 12.5, and 14 g/L.

To establish the model equations, a second-order polynomial equation, as shown in eq 1, was used.

1 $$y=\beta_{0}+\overset{3}{\underset{i=1}{\sum }}\beta_{i}X_{i}+\overset{3}{\underset{i=1}{\sum }}\beta_{ii}X_{i}^{2}+\overset{3}{\underset{\begin{array}{l} i=1\\ i<j \end{array}}{\sum }}\overset{3}{\underset{j=1}{\sum }}\beta_{ij}X_{i}X_{j}$$

In the model, *Y* denotes the response variable, β_0_ corresponds to the constant term, β_*i*_ represents the linear coefficients, β_*ii*_ refers to the quadratic coefficients, and β_*ij*_ describes the interaction effects between pairs of factors. The variables *X*_*i*_ and *X*_*j*_ indicate the independent factors included in the equation.

Bee products (organic bee pollen, organic bee bread/perga, and water-based organic propolis drops) were purchased as commercial products. Supplier documentation was used to report traceability information (e.g., harvest year and lot/batch number when available). The organic certificate for bee bread/perga reports harvest year (2025) and lot number AE261901. For bee pollen, the producer indicates production in the Mersin/Toros region and provides organic certification documentation. The propolis product is described as TURKGAP organic certified and formulated as water-based propolis (33% organic propolis) without alcohol.

The processing parameters for *P. spinosa* L.-based kombucha formulations fortified with propolis, pollen, and bee bread were optimized using a Box–Behnken Design (BBD) with three factors at three levels. The experimental plan comprised 15 runs in total. The adequacy of the model was evaluated using coefficients of determination (*R* ^2^ and adjusted *R* ^2^), lack-of-fit tests, one-way analysis of variance (ANOVA) results. For the preparation of blackthorn-propolis-enriched kombucha tea, RSM was also used to determine the optimal levels of *P. spinosa* L., tea, and propolis. The formulations were prepared with *P. spinosa* L. concentrations of 8%, 12%, and 16% (w/w); tea concentrations of 8, 11, and 14 g/L; and propolis concentrations of 1%, 1.5%, and 2% (v/v). RSM was used to determine the optimal concentrations of *P. spinosa* L. juice, tea, and pollen for the preparation of blackthorn-pollen-enriched kombucha tea. The formulations were prepared with *P. spinosa* L. concentrations of 8%, 12%, and 16% (w/w); tea concentrations of 8%, 11%, and 14% (w/w); and pollen concentrations of 3%, 5%, and 7% (w/w). The optimization of *P. spinosa* L., tea, and bee bread concentrations in the formulation of blackthorn-bee bread-enriched kombucha tea was performed using RSM. Kombucha formulations were prepared with *P. spinosa* L. juice at 8%, 12%, and 16% (w/w); tea at 8, 11, and 14 g/L; and bee bread at 2%, 5%, and 8% (w/w).

The addition levels of bee pollen, bee bread, and propolis were determined based on literature-supported concentration ranges and optimized using RSM, considering their distinct compositional and functional characteristics.

### 2.4 Sensory Analysis

Following the approach proposed by Yıkmış et al. (2020), and incorporating several modifications, the kombucha tea samples were subjected to sensory evaluation.^50^ A group of 15 semitrained assessors from Tekirdağ Namık Kemal University—each having prior instruction in sensory analysis methods—took part in the sensory assessment. Panel members were instructed to assess each sample on color, taste, smell, and general acceptance. Each sample was assigned a randomly selected three-digit alphanumeric identifier. Before beginning the sensory evaluation, panelists received instructions regarding the pretasting steps and the procedures to be followed during the assessment. To ensure accurate sensory perception, water was provided to the panelists for mouth rinsing and palate cleansing between tasting sessions. The sensory properties were evaluated using a nine-point hedonic scale (0–9). On this scale, the categories were defined as follows: 9 = excellent, 8 = very good, 7 = good, 6 = like slightly, 5 = neither like nor dislike, 4 = dislike slightly, 3 = dislike moderately, 2 = dislike, and 1 = dislike immensely. Scores of 6 or above were regarded as acceptable. Before the sensory assessment began, each participant completed a prescreening questionnaire to determine whether they had any allergies or sensitivities to bee-derived products such as propolis, pollen, or bee bread. Only individuals who indicated no previous allergic reactions to these substances were included in the sensory panel.

### 2.5 Total Phenolic Compounds (TPC)

The Folin–Ciocalteu assay was employed to quantify the TPC.^51^ For extract preparation, 2 mL of sample was mixed with 8 mL of 80% (v/v) methanol (1:5, v/v), vortexed, and centrifuged at 4000 rpm for 20 min to remove insoluble material and suspended particles. The clarified methanolic extract was used for analysis. Briefly, 50 μL of the clarified extract was mixed with 100 μL of Folin–Ciocalteu reagent (Sigma-Aldrich, F9252), and the volume was made up to 1450 μL with distilled water. After 10 min at room temperature, 50 μL of 7.5% (w/v) Na_2_CO_3_ was added (final volume 1500 μL). The mixture was incubated in the dark for 2 h, and absorbance was measured at 765 nm. TPC was expressed as mg gallic acid equivalents per 100 mL of sample (mg GAE/100 mL).

### 2.6 Total Flavonoid Content (TFC)

In this study, TFC was quantified using a colorimetric assay based on the procedure originally proposed by Zhishen et al.^52^ TFC was assessed using a colorimetric method in which appropriately diluted kombucha samples were successively treated with sodium nitrite (NaNO_2_), aluminum chloride (AlCl_3_), and sodium hydroxide (NaOH). The absorbance of the resulting solutions was recorded at 510 nm with a UV-Vis spectrophotometer, and the flavonoid concentration was reported as milligrams of catechin equivalents per liter (mg CE/L). All measurements were performed in triplicate

### 2.7 Antioxidant Activity (DPPH•)

The antioxidant activity was determined using the 2,2-diphenyl-1-picrylhydrazyl (DPPH•) radical scavenging assay.^53^ Briefly, 1 mL of kombucha sample was mixed with 1 mL of DPPH• solution (0.2 mM in methanol). The reaction mixture was incubated for 30 min at room temperature (25 ± 1 °C) in the dark. Absorbance was measured at 517 nm using a UV-Vis spectrophotometer (SP-UV/vis-300SRB, Spectrum Instruments, Melbourne, Australia). DPPH• radical scavenging activity was calculated using eq 2:

2 $$DPPH•radical\;scavenging\;activity\left(\%\right)=\left(A_{0}-A_{1}\right)/A_{0}\times 100$$

*A*_0_ represented the absorbance of the control sample, while *A*_1_ represented the absorbance measured for the kombucha tea samples.

### 2.8 Total Anthocyanin Content (TAC)

TAC was quantified using the pH differential assay.^54,55^ Prior to analysis, kombucha tea samples were diluted 1:10 (v/v) with distilled water and vortex-mixed thoroughly. The diluted samples were then centrifuged at 4000 rpm for 10 min to remove insoluble and suspended material, and the term “extract” refers to the clarified aqueous extract obtained after centrifugation (i.e., not phase separation). Subsequently, 1 mL of the clarified extract was brought to a final volume of 5 mL using 0.025 M potassium chloride buffer (pH 1.0). In parallel, another 1 mL aliquot of the clarified extract was diluted to 5 mL using 0.4 M sodium acetate buffer (pH 4.5). All prepared solutions were allowed to stand at room temperature for 15–30 min to equilibrate. Absorbance was measured at 515 and 700 nm using a UV-Vis spectrophotometer (SP-UV/vis-300SRB, Spectrum Instruments, Melbourne, Australia), with distilled water as the blank. Cyanidin-3-glucoside was used as the reference standard for quantifying monomeric anthocyanins, and TAC was calculated according to eq 3 Results were expressed as mg cyanidin-3-glucoside equivalents per liter (mg C_3_GE/L).

3 TAC(mg/L)=A(MW)(DF)1000/(ε)(L)

TAC: Total anthocyanin content

*A*: Absorbance difference, defined as the difference between measurements obtained at pH 1.0 and pH 4.5.

MW: Molecular weight of the anthocyanin standard (cyanidin-3-glucoside, 449.2 g/mol).

DF: Dilution factor

ε: Molar absorpsiyon katsayısı (26900)

*L*: Path length of the cuvette (cm)

Results were expressed as milligrams of cyanidin-3-glucoside equivalents per liter (mg C_3_GE/L).

### 2.9 Analysis of Phenolic Compounds (HPLC)

Chromatographic separation was carried out on an ACE Genix C18 column (250 × 4.6 mm, 5 μm; Agilent), following the procedure reported by Portu et al. (2017).^56^ Phenolics were characterized using an Agilent 1260 HPLC–DAD system operated under gradient conditions at 30 °C with a flow rate of 0.80 mL/min. The mobile phase comprised solvent A (water supplemented with 0.1% phosphoric acid) and solvent B, applied according to a predefined gradient program. Samples were injected at a volume of 10 μL, and chromatograms were recorded at 280, 320, and 360 nm. Quantitative results were reported as μg/mL.

Figure 1 shows phenolic chromatographic solutions.

**Figure 1:**
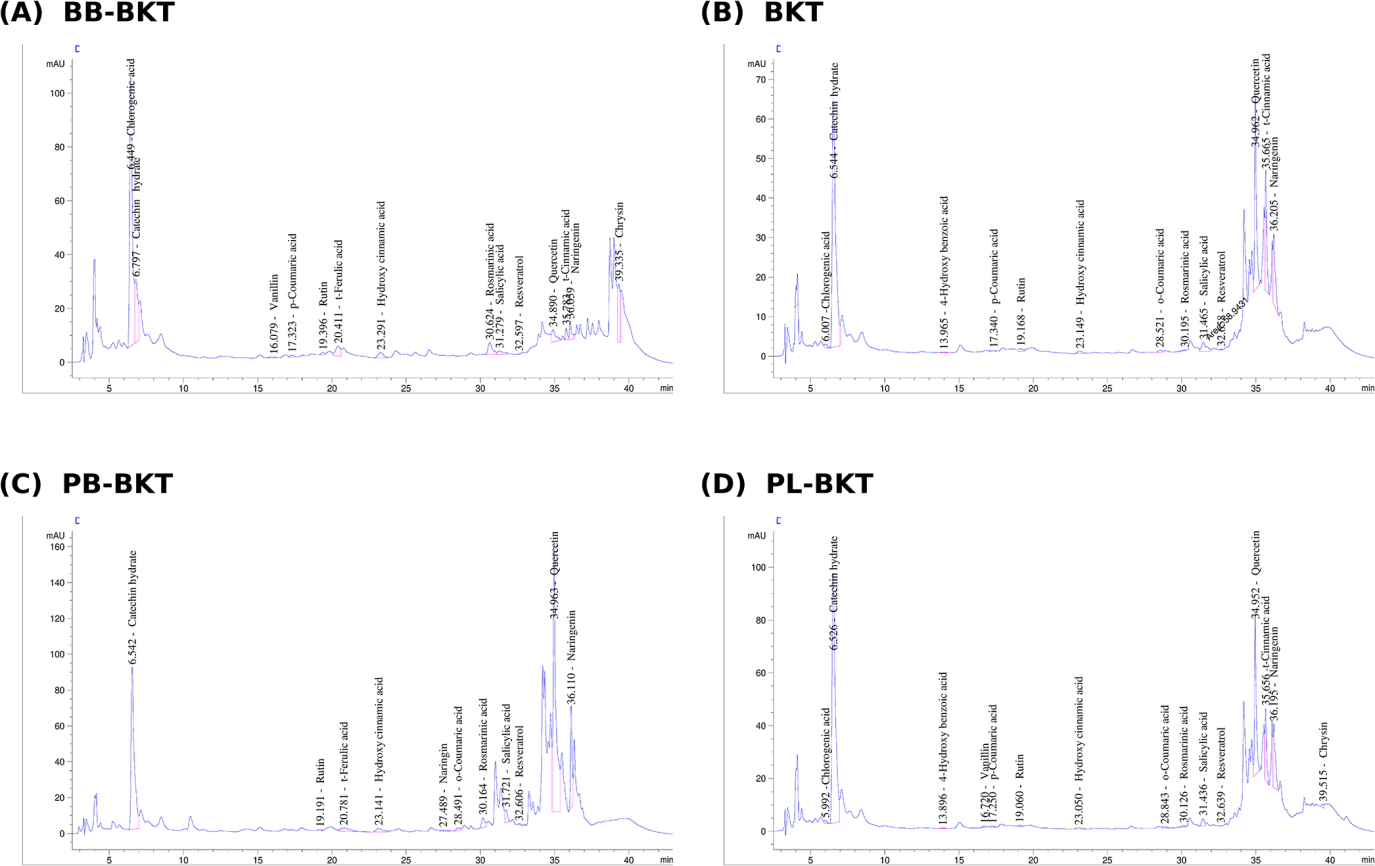
Phenolic compound chromatogram analysis.

### 2.10 Analysis of Organic Acids (HPLC)

HPLC was used to determine organic acid profiles, following a method adapted with minor modifications from Coelho et al.^57^ Organic acids were analyzed using an Agilent 1260 Infinity LC HPLC system fitted with refractive index (RID) and diode array (DAD) detectors. Samples were filtered through a 0.45 μm syringe filter, and a 20 μL aliquot was injected into an Agilent Hi-Plex H ion-exchange column (300 × 7.7 mm). Chromatographic separation was carried out using 10.0 mM H_2_SO_4_ as the mobile phase at a flow rate of 0.6 mL/min, with the column temperature set to 65 °C and the RID cell maintained at 35 °C. The total run time was 20 min. DAD additionally monitored tartaric, pyruvic, and acetic acids at 210 nm. Quantitative determination was carried out using external calibration standards, and the results were reported in g/L.

### 2.11 Determination of Free Amino Acids (LC–MS)

Amino acid composition was determined using a method adapted with minor modifications from the procedure described by Bilgin et al. (2019).^58^ A gradient elution scheme involving mobile phases A and B was applied to obtain chromatographic separation at a flow rate of 0.7 mL/min, yielding a total run duration of 7.5 min. Tandem mass spectrometric analyses were performed on an Agilent 6460 triple quadrupole LC–MS instrument (Agilent Technologies, Waldbronn, Germany) fitted with an electrospray ionization (ESI) interface. The mass spectrometer was operated with a gas temperature of 150 °C, a gas flow rate of 10 L/min, a nebulizer pressure of 40 psi, and a capillary voltage of +2000 V. All analyses were carried out in triplicate, and the results were reported as mg/100 mL.

### 2.12 Antidiabetic Activity

The antidiabetic activity of kombucha tea was investigated by determining its inhibitory capacity against α-glucosidase and α-amylase through a modified enzymatic assay.^59^ Absorbance readings were obtained using an SP-UV/Vis-300SRB UV-Vis spectrophotometer (Spectrum Instruments, Australia), and acarbose was used as the positive reference control in the antidiabetic assays.

### 2.13 Bioaccessibility Analysis

Samples were obtained according to the conditions defined during the production phase and gently shaken before analysis to ensure homogeneous distribution. For each digestion experiment, 5 mL of kombucha tea, corresponding to approximately 5 g, was measured and transferred to digestion vessels. Oral, gastric, and intestinal phases were applied sequentially, based on the previously reported standard static in vitro digestion protocol. All digestion steps were carried out at 37 ± 0.3 °C to simulate human gastrointestinal conditions.^60^

### 2.14 Statistical Analysis

Statistical analyses were performed using GraphPad Prism and SPSS software (version 20.0; SPSS Inc., Chicago, USA). Differences among samples were evaluated by ANOVA followed by Tukey’s post hoc multiple comparison test. RSM analyses were carried out using Minitab statistical software (version 18.1.1). For all statistical tests, differences were considered significant at *p* < 0.05.

## 3 Results and Discussion

### 3.1 Optimization of Sensory Analysis

The RSM method was used to optimize interval variables. The study was structured to examine both the open-loop and quadratic effects on the production of kombucha tea enriched with blackthorn, as well as formulations containing blackthorn combined with propolis, pollen, and bee bread. The two independent variables, blackthorn and black tea color, are given below for the production of kombucha tea with blackthorn eq 4, taste eq 5, smell eq 6, and general acceptance eq 7:

4 Color=4.50+1.114A−0.984B−0.01426AA+0.08115BB−0.05720AB

5 Taste=4.14+1.075A−0.905B−0.01406AA+0.07575BB−0.05500AB

6 Smell=4.718+0.2244A+0.0567B−0.02481AA−0.02750BB+0.04533AB

7 General Acceptance=5.93+0.1846A−0.103B−0.02680AA−0.02468BB+0.05297AB

The influence of three independent factors—blackthorn, black tea, and propolis—on the sensory attributes of color eq 8, taste eq 9, smell eq 10, and general acceptance eq 11 in blackthorn- and propolis-fortified kombucha tea production is presented below.

8 Color=−0.116+1.3143A−0.0514B−0.338C−0.03776AA+0.00325BB+0.421CC−0.00957AB−0.1706AC+0.0864BC

9 Taste=0.758+1.1656A+0.0292B−1.140C−0.034306AA−0.00132BB+0.5147CC−0.009017AB−0.13369AC+0.09307BC

10 Smell=0.364+1.1667A+0.0432B−1.147C−0.03354AA−0.00028BB+0.5505CC−0.01146AB−0.1338AC+0.0845BC

11 General Acceptance=−12.27+1.931A+0.879B+4.305C−0.06159AA−0.03060BB−1.072CC−0.01604AB−0.1400AC+0.0400BC

The impact of three independent variables—blackthorn, black tea, and pollen—on the sensory characteristics of color eq 12, taste eq 13, smell eq 14, and general acceptance eq 15 in pollen-enriched blackthorn kombucha tea is presented below.

12 Color=−0.042+1.2212A−0.0212B−0.0835C−0.03601AA+0.00330BB+0.02963CC−0.01031AB−0.04066AC+0.02074BC

13 Taste=0.994+1.0482A−0.0007B−0.2733C−0.03161AA+0.00065BB+0.03488CC−0.00848AB−0.03122AC+0.02327BC

14 Smell=0.166+1.0569A+0.0698B−0.1719C−0.03139AA−0.00282BB+0.02360CC−0.00977AB−0.03212AC+0.02627BC

15 General Acceptance=−10.98+1.7565A+0.931B+0.939C−0.05712AA−0.03121BB−0.0564CC−0.01371AB−0.03463AC−0.00179BC

The influence of three independent factors—blackthorn, black tea, and bee bread—on the sensory attributes of color eq 16, taste eq 17, smell eq 18 and general acceptance eq 19 in blackthorn- and bee bread-enriched kombucha tea production is summarized below.

16 Color=−0.064+1.1000A+0.0054B+0.0302C−0.034363AA+0.00331BB+0.01034CC−0.00954AB−0.02599AC+0.01338BC

17 Taste=0.100+1.1077A+0.1360B−0.0998C−0.034848AA−0.00372BB+0.01386CC−0.010403AB−0.022950AC+0.01920BC

18 Smell=−4.317+1.5001A+0.417B−0.0667C−0.05164AA−0.01847BB−0.00323CC−0.01085AB−0.02148AC+0.03115BC

19 General Acceptance=−3.998+1.3623A+0.5139B+0.0835C−0.04474AA−0.01899BB−0.00171CC−0.01274AB−0.02282AC+0.01570BC

ANOVA results indicate that the model for use in blackthorn kombucha tea is highly reliable and significant. The use of a response surface algorithm comprehensively integrated the distribution of fermentation in Himalayan black tea beverages, similar to our study. The model’s high *R* ^2^ values and lack of fit indicate robustness to the design process. The model’s robustness was confirmed by ANOVA, demonstrating its robustness across all investigated effects (*p* < 0.001).^61^ When the differences between the RSM optimization results and the experimental data in Table S1 were examined, it was determined that the deviations for color (0.48%), taste (3.34%), smell (0.94%), and general acceptance (1.07%) were relatively low. These low differences clearly demonstrate the model’s strong predictive ability.

In the optimization results, classification against sensitive data was observed in color (2.42%), taste (5.11%), smell (4.84%), and general acceptance (5.65%) parameters, with differences remaining at 6% (Table S2). Propolis (*X*_3_) was found to have a substantial effect on taste (*F* = 133.45), while the squared effect of blackthorn (*X*_1_^2^) made the most significant contribution to the model with *F* = 2383.45. The model’s *R* ^2^ values ranged from 98.73% to 99.88%, indicating extremely high predictive accuracy. These data support the effective role of propolis amount in increasing the product’s sensory flexibility and the robustness of the model. It was observed that sensory parameters improved as a result of propolis addition to yellow cherry juice (*Prunus avium* L.) prepared by Yıkmış et al. (2025).^62^ Poppy (*Papaver rhoeas* L.) sherbet showed lower color and general acceptance scores due to propolis addition and ultrasound application. According to this study, both propolis addition and ultrasound-dispersed poppy sherbet increased the acids, and the loss of color vibrancy can be attributed to.^63^

Blackthorn-pollen and blackthorn-bee bread combinations also stood out with high agreement and low error rates. In a study, RSM was used to produce pollen-supplemented kombucha tea. Consistent with our findings, the ANOVA results showed that the model incorporating the three factors (SCOBY, pollen and sodium selenite) and their interactions was statistically significant (*p* < 0.05).^64^ The experimental and optimization differences were calculated as 3.17% for color, 7.22% for taste, 6.63% for smell, and 3.81% for general acceptance (Table S3). For the bee bread variant, the differences were 0.42%, 1.53%, 0.55%, and 0.95%, respectively, demonstrating near-perfect predictive accuracy (Table S4).

As shown in Table S5, the *p*-values for all sensory parameters in the model (color, taste, smell, and general acceptance) were below 0.001, indicating statistical significance (ANOVA tables). Specifically, the flavor ratio (*X*_1_) was determined to be the most influential variable for the smell parameter, with an *F*-value of 745.06. The amount of black tea (*X*_2_) had a particularly significant effect on color and taste. The model’s *R* ^2^ values ranged from 98.87% to 99.42%, indicating that it explained the data with high accuracy. The results from the Blackthorn-propolis interpretation (Table S6) support the model’s high explanatory power and significance (ANOVA tables). The *F*-values of the model for color, taste, and smell parameters were calculated as 94.01, 475.67, and 107.13, respectively, and were significant at the total *p* < 0.001 level.

According to the data in Tables S7 and S8, *R* ^2^ values were above 99% in both models. The high *F*-values of the interaction terms *X*_1_^2^ and *X*_1_ *X*_3_ (608.82 and 160.85) in the pollen-supplemented kombucha samples indicate the synergy between blackthorn and pollen ratios. These results indicate that the addition of bee bread, in particular, increases sensory liking, and that the ideal formulation for blackthorn-based kombucha production is approximately 12–13% blackthorn, 11–13 g/L black tea, and 8% bee bread. The fact that the differences between the experimental and predicted values generally remain within 1–7% demonstrates that the RSM approach is a highly reliable method for optimizing blackthorn-based functional beverages.

### 3.2 Sensory Analysis

Panelists evaluated the teas for color, taste, smell, sourness and general acceptance. According to the sensory analysis, blackthorn (*P. spinosa* L.) and bee bread-enriched kombucha tea received the highest general acceptance score (8.32), showing a statistically significant difference from all other groups (*p* < 0.05). In addition, this sample was particularly noteworthy for its high taste score (8.25). Considering the contribution of bee bread to both sensory and bioactive properties during the fermentation process, the diversity of volatile aromatic compounds plays a critical role.^65^ When evaluated for visual quality, the blackthorn kombucha tea sample had the highest mean score (8.13), indicating that this formulation was highly appreciated by the panelists for its color.

In the smell assessment, the blackthorn-pollen-enriched sample achieved the highest mean score (8.21), indicating a rich, well-developed aromatic profile. The presented radar chart clearly illustrates how all formulations performed across different sensory attributes. In line with our findings, pollen-enriched biscuits have also been associated with high scores for aroma persistence in previous studies. The incorporation of pollen enhanced the perception of floral, fruity, and herbal aromatic notes, contributing positively to the sensory profile.^66^ Regarding the sourness parameter, the blackthorn-pollen-enriched kombucha tea sample stood out with a mean score of 8.19, suggesting that kombucha formulations with relatively higher acidity may be preferred within certain sensory thresholds. Conversely, the blackthorn-propolis-enriched kombucha tea sample received lower mean scores across all sensory attributes. In a previous study, the addition of propolis to yogurt similarly decreased sensory acceptability, in agreement with the findings of the present study.^67^ Following the addition of propolis to poppy sherbet, lower scores were observed for both taste and general acceptance. This decline can be attributed to the acidic taste imparted by propolis, which may have negatively influenced consumer perception.^63^ The sensory scores of all samples, ranging between 7.30 and 8.32, indicate that they were generally accepted by the semitrained panelists, demonstrating the success of the optimized formulations. The blackthorn-bee bread-enriched kombucha tea formulation stands out for balanced taste and general acceptance, whereas the blackthorn-pollen-enriched kombucha tea can be considered a strong alternative, particularly for aroma and sourness attributes. Figure 2 presents the radar chart.

**Figure 2:**
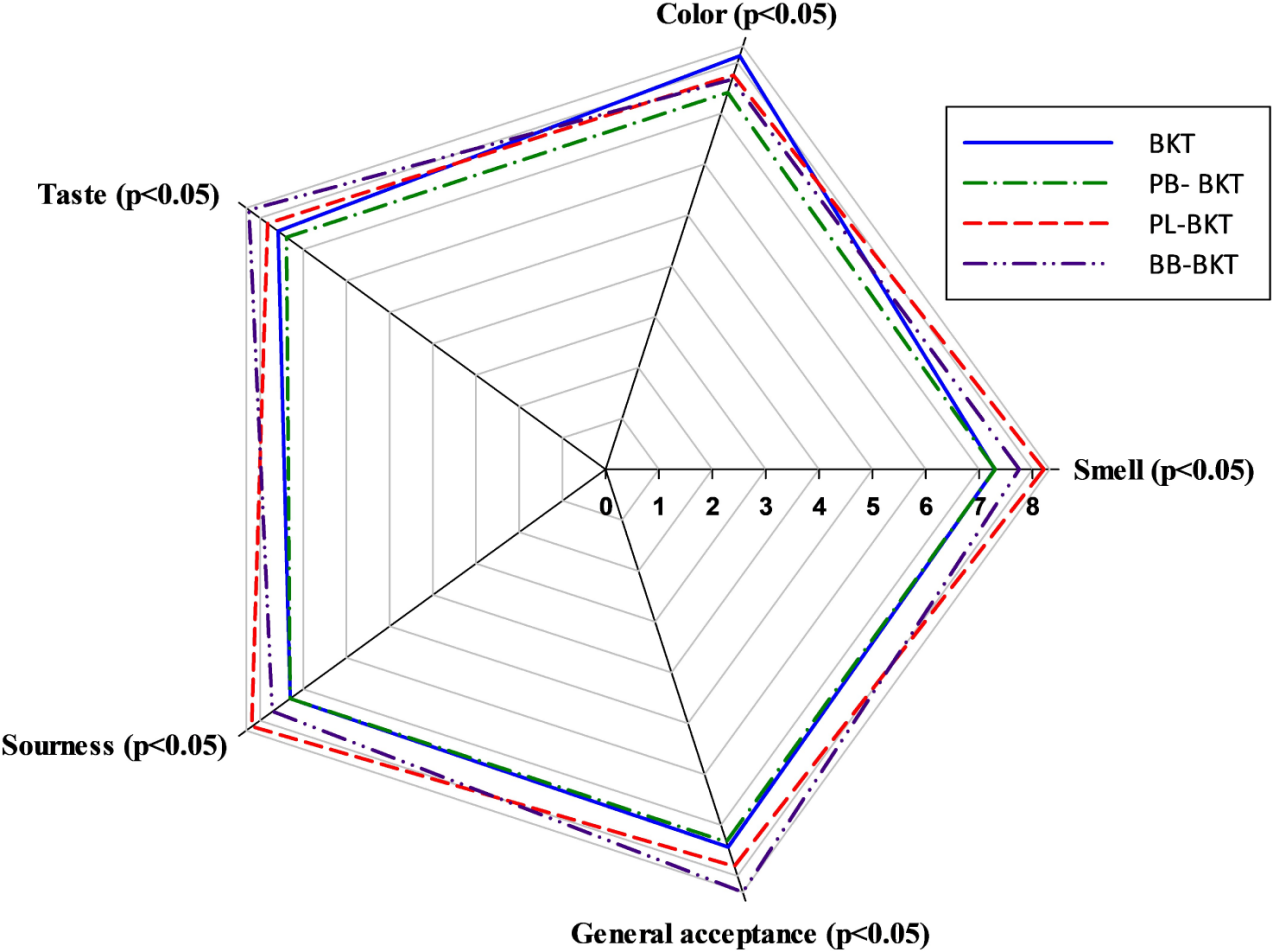
Radar plot of sensory scores (9-point hedonic scale) for control blackthorn kombucha tea (BKT) and propolis- (PB-BKT), pollen- (PL-BKT), and bee bread-enriched (BB-BKT) blackthorn kombucha teas.

### 3.3 Bioactive Compounds

According to the graph, significant differences in TPC and TFC content were observed among blackthorn kombucha samples enriched with bee products (*p < 0.05). The BB-BKT sample enriched with bee bread showed the highest TPC of all groups, at 257.10 mg GAE/100 mL. This increase may be due to the abundance of phenolics in bee bread and to greater liberation (or release) of bound phenolics during fermentation. Widely accepted as a food additive, fermentation allows for the discovery and inclusion of new probiotics in the human diet, thereby contributing positively to health outcomes.^14^ A similar trend was observed in flavonoid content; the BB-BKT sample was found to be statistically higher than the other samples with a value of 36.16 mg CE/100 mL. This indicates that the addition of bee bread significantly increased the biologically active compound content of the kombucha samples and enriched the phenolics profile (Figure 2). Consistent with the findings reported here, a separate study also observed a statistically significant elevation in the total flavonoid content of kiwi juice samples fortified with bee bread.^68^ According to the DPPH• results, the BB-BKT sample showed the highest radical scavenging activity (57.05 μmol TE/mL), and this difference was statistically significant (*p* < 0.01). Studies on bee bread reported previously have shown the same thing.^69,70^ Additionally, pollen fermentation mimics naturally evolved processes aimed at enhancing nutritional value. In the hive, pollen is fermented and stored in honey-enriched, wax-sealed combs, resulting in bee bread, which combines pollen-derived phytochemicals with improved bioaccessibility and bioactive compounds formed during fermentation.^71^ As a result of this combination, bee bread contains higher levels of essential amino acids and vitamins (e.g., B and K groups), as well as an increased content of bioactive polyphenols.

Comparing the samples showed no significant variation in TAC content (*p* > 0.05), indicating that anthocyanins were more stable during fermentation. TPC and TAC showed a clear relationship (*r* > 0.90). In conclusion, the graph shows that adding bee bread enhances kombucha’s functional properties, particularly increasing antioxidant activity (Figure 3).

**Figure 3:**
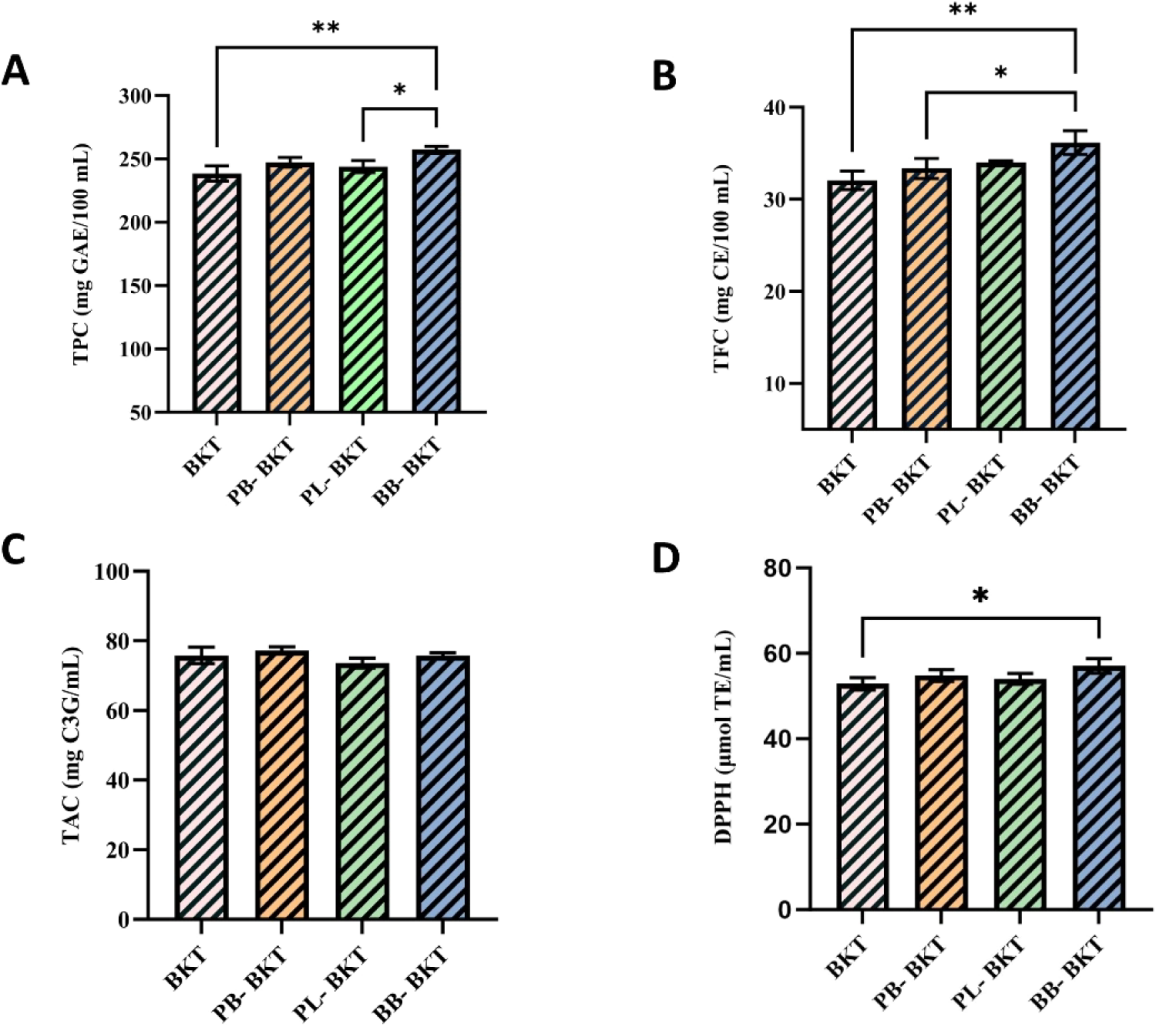
TPC (A), TFC (B), TAC (C), and DPPH• (D) results of kombucha tea samples. BKT: Blackthorn kombucha tea; PB: Propolis blackthorn kombucha tea; PL: Pollen blackthorn kombucha tea; BB: Bee bread blackthorn kombucha tea. TPC: Total phenolic content; TFC: Total flavonoid content; TAC: Total anthocyanin content; DPPH•: 2,2-Diphenyl-1-picrylhydrazyl; GAE: Gallic acid equivalent; CE: Catechin equivalent; C_3_G: Cyanidin-3-glucoside; TE: Trolox equivalent. All analyses were conducted in duplicate (*p < 0.05; **p < 0.01).

### 3.4 Phenolic Compounds (HPLC)

As presented in Table 1, the total quantified phenolic content differed significantly among the treatments (*p* < 0.05). The control sample (BKT) exhibited the lowest phenolic concentration (106.35 ± 2.25 μg/mL), whereas pollen supplementation (PL-BKT) resulted in a substantial increase, reaching 159.23 ± 4.82 μg/mL. Further enhancement of the phenolic pool was achieved through propolis (PB-BKT: 178.66 ± 5.04 μg/mL) and bee bread (BB-BKT: 186.15 ± 0.97 μg/mL) supplementation. No statistically significant difference was observed between PB-BKT and BB-BKT in total quantified phenolics; however, both treatments exhibited significantly higher values than PL-BKT and the control, indicating a more pronounced phenolic enrichment.

**Table 1:** Changes in Total Phenolic Compounds of Kombucha Samples Subjected to BKT, PB-BKT, PL-BKT and BB-BKT[Table-fn tbl1fn1]

Phenolic compound (μg/mL)	BKT	PB-BKT	PL-BKT	BB-BKT
Chlorogenic acid	0.63 ± 0.01^a^	0.00 ± 0.00^a^	0.78 ± 0.02^a^	79.04 ± 4.66^b^
Catechin hydrate	77.74 ± 1.67^b^	101.72 ± 2.19^c^	121.2 ± 3.36^d^	24.35 ± 0.68^a^
Caffeic acid	nd	nd	nd	nd
4-Hydroxy benzoic acid	0.00 ± 0.00	0.00 ± 0.00	0.00 ± 0.00	0.00 ± 0.00
Vanillin	0.00 ± 0.00^a^	0.00 ± 0.00^a^	0.33 ± 0.03^c^	0.13 ± 0.01^b^
*p-*Coumaric acid	0.00 ± 0.00^a^	0.00 ± 0.00^a^	0.11 ± 0.01^b^	00.21 ± 0.02^c^
Rutin	0.55 ± 0.01^c^	0.23 ± 0.01^b^	00.03 ± 0.00^a^	0.75 ± 0.06^d^
*t*-Ferulic acid	0.00 ± 0.00^a^	2.82 ± 0.08^b^	0.00 ± 0.00^a^	2.73 ± 0.22^b^
Hydroxy cinnamic acid	0.50 ± 0.01^a^	1.50 ± 0.04^c^	0.53 ± 0.01^a^	1.25 ± 0.10^b^
Naringin	0.00 ± 0.00^a^	0.88 ± 0.03^b^	0.00 ± 0.00^a^	0.00 ± 0.00^a^
*o-*Coumaric acid	0.17 ± 0.00^b^	0.53 ± 0.02^c^	0.2 ± 0.02^b^	0.00 ± 0.00^a^
Rosmarinic acid	0.17 ± 0.00^a^	2.45 ± 0.05^b^	0.41 ± 0.01^a^	3.05 ± 0.24^c^
Salicylic acid	2.32 ± 0.08^b^	3.34 ± 0.17^c^	1.42 ± 0.04^a^	1.55 ± 0.12^a^
Resveratrol	0.01 ± 0.00^a^	0.29 ± 0.01^c^	0.02 ± 0.00^b^	0.01 ± 0.00^a^
Quercetin	10.45 ± 0.15^b^	47.32 ± 2.07^c^	13.18 ± 0.37^b^	2.87 ± 0.23^a^
*t*-Cinnamic acid	6.76 ± 0.15^d^	0.00 ± 0.00^a^	5.91 ± 0.16^c^	1.04 ± 0.08^b^
Naringenin	7.04 ± 0.15^b^	17.59 ± 0.38^d^	10.51 ± 0.64^c^	2.75 ± 0.08^a^
Chrysin	0.00 ± 0.00^a^	0.00 ± 0.00^a^	4.54 ± 0.13^b^	66.42 ± 1.84^c^
Flavones	nd	nd	nd	nd
Total quantified	106.35 ± 2.25^a^	178.66 ± 5.04^c^	159.23 ± 4.82^b^	186.15 ± 0.97^c^

i Data are presented as mean ± standard deviation (*n* = 3). Values denoted by different letters within the same row indicate statistically significant differences (*p* < 0.05). BKT: Blackthorn kombucha tea; PB-BKT: Propolis blackthorn kombucha tea; PL-BKT: Pollen blackthorn kombucha tea; BB-BKT: Bee bread blackthorn kombucha tea. n.d.: could not be detected.

The compound-level distribution suggests that the observed elevation in total phenolics is driven by selective, matrix-dependent enrichment. Bee bread is a functional food thanks to its bioactive compounds and chemical diversity.^72^ The marked increase in chlorogenic acid concentration in BB-BKT, to 79.04 ± 4.66 μg/mL, was a key contributor to the elevated total phenolic content in this group. In contrast, chlorogenic acid remained undetectable or present at negligible levels in the other treatments. Likewise, the enrichment of thermosonicated kiwi juice with bee bread resulted in a statistically significant elevation in chlorogenic acid levels.^68^ The highest catechin hydrate concentration was observed in PL-BKT (121.2 ± 3.36 μg/mL), indicating that pollen supplementation preferentially enhances the flavan-3-ol fraction. In contrast, the markedly elevated levels of quercetin (47.32 ± 2.07 μg/mL) and naringenin (17.59 ± 0.38 μg/mL) detected in PB-BKT support the flavonoid-enriching effect of propolis (*p* < 0.05). In another study, propolis supplementation of thermosonicated yellow cherry juice was found to increase quercetin content significantly. Additionally, the exclusive emergence of chrysin in the enriched samples, particularly its pronounced accumulation in BB-BKT (66.42 ± 1.84 μg/mL), suggests that bee bread supplementation can substantially reshape the specific phenolic signature of the matrix. This interpretation is further supported by the highest rosmarinic acid level observed in BB-BKT (3.05 ± 0.24 μg/mL). Conversely, the absence of caffeic acid and the flavones fraction across all treatments (n.d.) indicates that the applied interventions exerted limited effects on specific phenolic subclasses.

### 3.5 Free Amino Acids (LC–MS)

The PL-BKT sample, which was supplemented with pollen, exhibited the highest free amino acid content (see Table 2). This outcome differed significantly from all other groups (*p* < 0.05). Significant increases were observed in proline (47.37 mg/100 mL), leucine (4.10 mg/100 mL), and lysine (4.54 mg/100 mL), indicating that pollen, due to its high protein content, increases amino acid release during fermentation. In contrast, amino acid levels were lower in the sample supplemented with propolis alone (PB-BKT), and some amino acids (e.g., glutamic acid, histidine, threonine) were not statistically different from the BKT sample (*p* > 0.05).

**Table 2:** Effects of BKT, PB-BKT, PL-BKT and BB-BKT on Free Amino Acids (mg/100 mL)[Table-fn tbl2fn1]

		Samples			
	Analyses	BKT	PB-BKT	PL-BKT	BB-BKT
Amino acid content (mg/100 mL)	Alanine	0.13 ± 0.00^a^	0.06 ± 0.00^a^	4.09 ± 0.11^c^	0.57 ± 0.01^b^
	Arginine	0.07 ± 0.05^a^	0.01 ± 0.00^a^	2.49 ± 0.01^b^	0.09 ± 0.00^a^
	Aspartic Acid	0.00 ± 0.00^a^	0.00 ± 0.00^a^	1.60 ± 0.01^c^	1.47 ± 0.02^b^
	Cystine	0.00 ± 0.00^a^	0.00 ± 0.00^a^	0.03 ± 0.01^b^	0.00 ± 0.00^a^
	Glutamic Acid	0.14 ± 0.00^a^	0.06 ± 0.01^a^	3.07 ± 0.11^c^	0.89 ± 0.02^b^
	Glycine	0.00 ± 0.00^a^	0.17 ± 0.01^b^	3.56 ± 0.05^d^	0.30 ± 0.01^c^
	Histidine	0.05 ± 0.01^a^	0.02 ± 0.00^a^	1.22 ± 0.01^c^	0.37 ± 0.00^b^
	Isoleucine	0.01 ± 0.00^a^	0.01 ± 0.00^a^	0.86 ± 0.01^c^	0.13 ± 0.02^b^
	Leucine	0.07 ± 0.01^a^	0.07 ± 0.01^a^	4.10 ± 0.11^c^	0.35 ± 0.02^b^
	Lysine	0.15 ± 0.00^a^	00.10 ± 0.00^a^	4.54 ± 0.13^b^	0.24 ± 0.01^a^
	Methionine	0.03 ± 0.00^a^	0.02 ± 0.00^a^	1.20 ± 0.01^c^	0.09 ± 0.01^b^
	Ornithine	0.05 ± 0.00^a^	0.07 ± 0.01^a^	1.29 ± 0.01^c^	0.14 ± 0.01^b^
	Phenylalanine	0.04 ± 0.01^a^	0.02 ± 0.00^a^	2.64 ± 0.08^b^	0.19 ± 0.00^a^
	Proline	1.08 ± 0.01^a^	2.34 ± 0.01^b^	47.37 ± 0.31^d^	18.71 ± 0.18^c^
	Serine	0.04 ± 0.01^a^	0.03 ± 0.01^a^	1.50 ± 0.01^c^	0.37 ± 0.01^b^
	Threonine	0.06 ± 0.01^a^	0.01 ± 0.00^a^	1.42 ± 0.03^c^	0.29 ± 0.01^b^
	Tyrosine	0.02 ± 0.00^a^	0.02 ± 0.00^a^	1.90 ± 0.03^c^	0.23 ± 0.02^b^
	Valine	0.02 ± 0.00^a^	0.00 ± 0.00^a^	1.74 ± 0.01^c^	0.23 ± 0.01^b^
	Taurine	0.05 ± 0.00^a^	0.04 ± 0.00^a^	0.05 ± 0.01^a^	0.05 ± 0.01^a^

i The mean value, along with the standard deviation, is given for the data (*n* = 3). Values indicated by different letters within the same row differ significantly (*p* < 0.05). BKT: Blackthorn kombucha tea; PB-BKT: Propolis blackthorn kombucha tea; PL-BKT: Pollen blackthorn kombucha tea; BB-BKT: Bee bread blackthorn kombucha tea.

Fermentation is a chemical process where microbes improve the body’s access to nutrients in bee pollen.^73^ The nutritional and functional value of pollen is enriched because microbial metabolic activity promotes the synthesis or proliferation of key bioactive compounds, including amino acids, vitamins and enzymes.^74^ By exploiting the activity of specific enzymes, enzymatic hydrolysis serves as an effective approach for breaking down complex bee pollen macromolecules, such as proteins and carbohydrates, into smaller, more bioaccessible components, including essential amino acids and monosaccharides. This increase in the release and availability of bioactive compounds and nutrients offers potential health advantages to the consumer.^75^ The incorporation of pollen into fermented kombucha tea led to an increase in its amino acid content. This enhancement is attributed to the intensified release of free amino acids driven by proteolytic activity during fermentation, resulting in an amino acid profile that may be considered functionally relevant.^76^

The bee bread supplemented sample (BB-BKT), while generally lower than PL-BKT, showed significant increases in many amino acids compared to BKT and PB-BKT (*p* < 0.05). Specifically, the increased values of proline (18.71 mg/100 mL) and glutamic acid (0.89 mg/100 mL) reflect the enriching effect of bee bread on the fermentation microflora. Conversely, the similar levels of taurine in all samples (*p* > 0.05) suggest that this compound is stable during fermentation. It can be said that the addition of pollen and bee bread significantly enhances the nutritional quality of the product by improving the free amino acid profile.

### 3.6 Antidiabetic Activity

Inhibiting the enzymes integral to carbohydrate digestion, namely α-glucosidase and α-amylase, is viewed as a viable treatment for type 2 diabetes. This approach delays or prevents carbohydrate hydrolysis, subsequently limiting postmeal glucose production and uptake.^77^

As shown in Figure 3, the PB-BKT sample exhibited the highest value (40.7 ± 1.32%) in α-glucosidase inhibitory activity, significantly distinguishing it from the control group (BKT: 33.27 ± 1.23%; *p* < 0.01) and the other supplemented samples (PL-BKT: 31.85 ± 1.16%; BB-BKT: 34.33 ± 0.71%; *p* < 0.05). This difference clearly demonstrates the inhibitory effect of propolis addition on enzyme activity and statistically supports the increase in the antidiabetic capacity of the formulation. The high inhibitory rate of PB-BKT suggests that the phenolic and flavonoid components of propolis reduce α-glucosidase activity, slowing down the rate of carbohydrate hydrolysis and potentially improving glycemic control. These results are similar to previous reports showing inhibition of α-amylase and α-glucosidase in Nigerian propolis,^78^ Moroccan propolis^79^ Turkish propolis^80^ and Australian propolis^77^ and concluding that phenolic compounds and particularly flavonoids can inhibit these digestive enzymes, which may delay the digestion of carbohydrates and prevent glucose absorption, thereby reducing postprandial hyperglycemia.

Similarly, the results regarding α-amylase inhibitory activity (Figure 4) also confirm the superiority of PB-BKT. The value of 42.02 ± 1.78% recorded in this sample is significantly higher than both the control group (BKT: 35.88 ± 0.73%; *p* < 0.01) and the PL-BKT (35.71 ± 0.60%) and BB-BKT (36.84 ± 1.17%) samples (*p* < 0.05). These results indicate that propolis produces a strong inhibition on both enzyme systems, and the PB-BKT formulation provides the highest antidiabetic activity. The obtained data scientifically support the possibility of propolis as a natural antidiabetic agent in functional food compositions.

**Figure 4:**
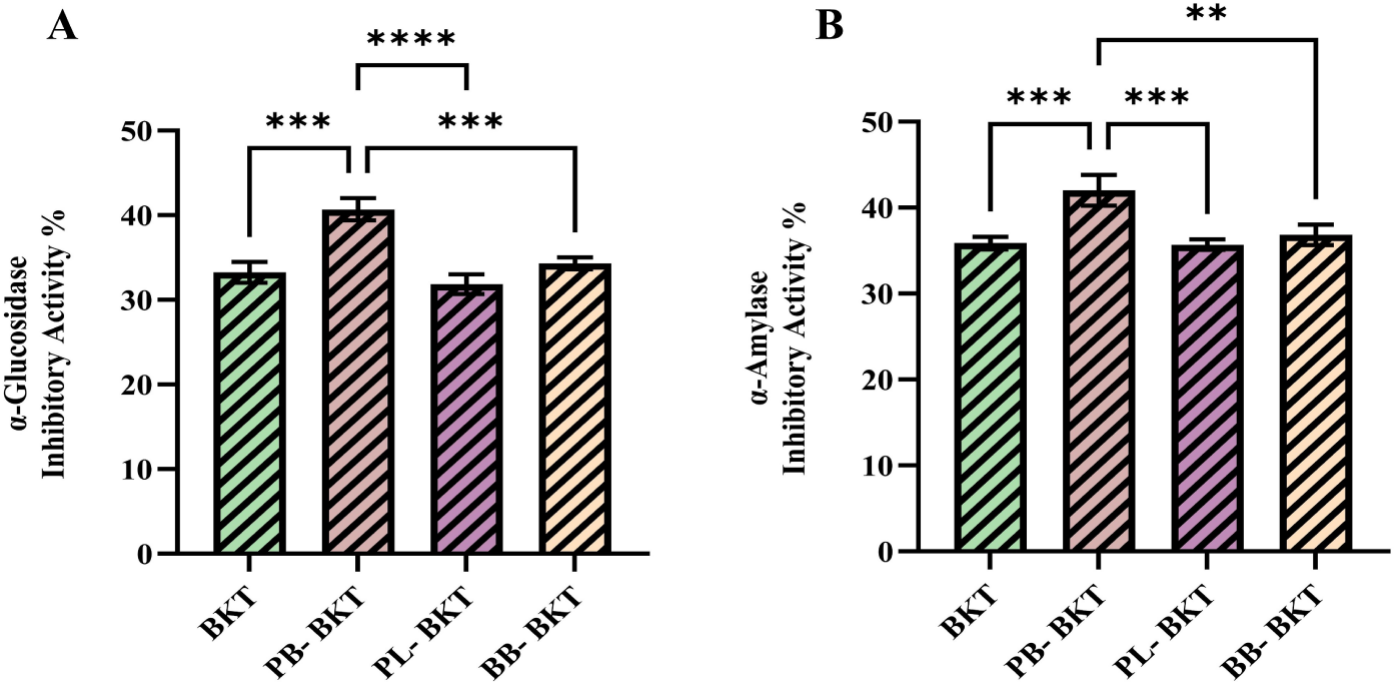
α-glucosidase inhibitory activity % (A) and α-amylase inhibitory activity % (B) of BKT, PB-BKT, PL-BKT, and BB-BKT. BKT: Blackthorn kombucha tea; PB-BKT: Propolis blackthorn kombucha tea; PL-BKT: Pollen blackthorn kombucha tea; BB-BKT: Bee bread blackthorn kombucha tea. Different letters within the same row indicate statistically significant differences (* *p* < 0.05; ** *p* < 0.01; *** *p* < 0.001; **** *p* < 0.0001).

### 3.7 Bioaccessibility

According to the data presented in Table 3, enrichment of blackthorn kombucha with different bee products significantly affected TPC, TFC, TAC, and DPPH• levels during the digestion simulation. Specifically, in the undigested samples, the BB-BKT group (bee bread blackthorn kombucha) was observed to have the highest phenolic and flavonoid contents, with 257.10 mg GAE/100 mL TPC and 36.16 mg CE/100 mL TFC. This result suggests that bee products (especially bee bread) increase antioxidant capacity by enriching the phenolics (*p* < 0.05). Furthermore, when DPPH• values were examined, the BB-BKT sample reached the highest radical scavenging activity with 57.05 μmol TE/mL (Table 3). This finding reinforces the recognition of bee bread as a rich and potent source of bioactive compounds.^81^

**Table 3:** TPC, TFC, TAC and DPPH• during Simulated Digestion in BKT, PB-BKT PL-BKT and BB-BKT Samples[Table-fn tbl3fn1]

Phases	Samples	TPC (mg GAE/100 mL)	TFC (mg CE/100 mL)	TAC (mg C_3_G/mL)	DPPH• (μmol TE/mL)
Undigested	BKT	238.42 ± 6.10^a^	32.06 ± 1.00^a^	75.83 ± 2.35^a^	52.87 ± 1.45^a^
	PB-BKT	247.24 ± 3.83^ab^	33.35 ± 1.10^a^	77.25 ± 1.05^a^	54.79 ± 1.35^ab^
	PL-BKT	243.91 ± 4.76^a^	34.02 ± 0.16^ab^	73.64 ± 1.40^a^	54.04 ± 1.25^ab^
	BB-BKT	257.10 ± 2.74^b^	36.16 ± 1.28^b^	75.79 ± 0.74^a^	57.05 ± 1.69^b^
Oral digestion	BKT	197.89 ± 5.06^a^	26.93 ± 0.85^a^	62.94 ± 1.95^a^	44.94 ± 1.24^a^
	PB-BKT	203.30 ± 6.05^a^	27.74 ± 0.87^ab^	63.75 ± 1.82^a^	46.39 ± 1.21^ab^
	PL-BKT	201.89 ± 3.87^a^	28.12 ± 0.74^ab^	63.45 ± 2.11^a^	45.96 ± 1.11^ab^
	BB-BKT	208.48 ± 1.32^a^	30.31 ± 1.49^b^	63.05 ± 0.57^a^	48.63 ± 1.11^b^
Gastric digestion	BKT	100.93 ± 2.58^a^	18.58 ± 0.58^a^	40.91 ± 1.26^a^	28.76 ± 0.79^a^
	PB-BKT	105.00 ± 1.25^ab^	19.16 ± 0.57^a^	41.85 ± 0.84^a^	29.69 ± 0.77^a^
	PL-BKT	104.99 ± 2.07^ab^	19.40 ± 0.51^a^	41.85 ± 0.44^a^	29.4 ± 0.67^a^
	BB-BKT	107.35 ± 1.72^b^	20.01 ± 0.63^a^	42.02 ± 0.65^a^	30.08 ± 1.42^a^
Intestinal digestion	BKT	50.28 ± 1.00^a^	9.16 ± 0.43^a^	20.45 ± 0.63^a^	13.8 ± 0.38^a^
	PB-BKT	53.89 ± 1.19^b^	9.90 ± 0.50^ab^	20.93 ± 0.42^a^	15.44 ± 0.54^a^
	PL-BKT	53.49 ± 0.93^b^	9.70 ± 0.26^ab^	20.84 ± 0.34^a^	14.11 ± 0.33^a^
	BB-BKT	56.75 ± 1.09^c^	10.67 ± 0.86^b^	21.01 ± 0.32^a^	15.44 ± 1.33^a^
Recovery %	BKT	21.09 ± 0.15^a^	28.57 ± 0.46^a^	26.97 ± 0.01^a^	26.11 ± 0.01^a^
	PB-BKT	21.80 ± 0.64^a^	29.69 ± 0.65^a^	27.09 ± 0.20^ab^	28.18 ± 0.74^a^
	PL-BKT	21.94 ± 0.65^a^	28.53 ± 0.80^a^	28.31 ± 0.87^b^	26.11 ± 0.01^a^
	BB-BKT	22.07 ± 0.20^a^	29.56 ± 3.20^a^	27.73 ± 0.45^ab^	27.12 ± 3.09^a^

i Results are presented as mean ± standard deviation (*n* = 3). Values denoted by different letters within the same row indicate statistically significant differences (*p* < 0.05). BKT: Blackthorn kombucha tea; PB-BKT: Propolis blackthorn kombucha tea; PL-BKT: Pollen blackthorn kombucha tea; BB-BKT: Bee bread blackthorn kombucha tea; TPC: Total phenolic content; TFC: Total flavonoid content; TAC: Total anthocyanin content; DPPH•: 2,2-Diphenyl-1-picrylhydrazyl; GAE: Gallic acid equivalent; CE: Catechin equivalent; C_3_G: Cyanidin-3-glucoside; TE: Trolox equivalent.

As the digestive phases progressed, a gradual decrease in the levels of bioactive compounds occurred in all blackthorn kombucha samples, as shown in Table 3. In the gastric phase, TPC and TFC values decreased by almost half, and in the intestinal phase, they decreased to approximately 20–30% of their initial values. This decrease is due to pH changes and enzymatic hydrolysis processes in the digestive environment, which reduce the stability of phenolic compounds. Another study, similar to ours, observed a significant and gradual decrease in TFC levels as the digestive phases progressed.^82^ A different study on TFC in various food matrices has found a similar result.^83^ However, the postdigestion phenolic and flavonoid levels of the PB-BKT and BB-BKT groups remained higher than those of the BKT, suggesting that the phenolic compounds of these bee products exert a protective effect against degradation during digestion. The elevated phenolic content found in bee bread, relative to bee pollen, is explainable by the partial breakdown of the pollen grain’s multilayered structure, a result of bacterial enzymatic action during the bee bread fermentation.

These findings are also supported by the recovery percentages calculated at the end of the intestinal phase. As seen in Table 3, the BB-BKT sample had the highest recovery rates for TPC (22.07%) and TFC (29.56%). This result indicates that the bee bread addition not only increased the initial phenolic content but also enhanced postdigestion bioaccessibility. It was concluded that blackthorn kombucha enriched with bee products maintained the stability of bioactive compounds throughout the digestion process, with the addition of bee bread being the component that most supported the bioaccessibility of phenolic compounds.

### 3.8 Chemometric Evaluation of Blackthorn Kombucha Tea Formulations: PCA and Pearson Correlation

The Pearson correlation heatmap presented in Figure 5 supports the sample discrimination observed in the PCA by elucidating the strength and direction of intervariable relationships. In particular, the sensory attribute general acceptance exhibited a very strong positive correlation with taste (*r* = 0.992). Similarly, high positive correlations were identified between general acceptance and *p*-Coumaric acid (*r* = 0.994), total flavonoid content (TFC; *r* = 0.917), chrysin (*r* = 0.915), and chlorogenic acid (*r* = 0.891). In contrast, pronounced negative associations were observed with *o*-Coumaric acid (*r* = −0.806) and salicylic acid (*r* = −0.790).

**Figure 5:**
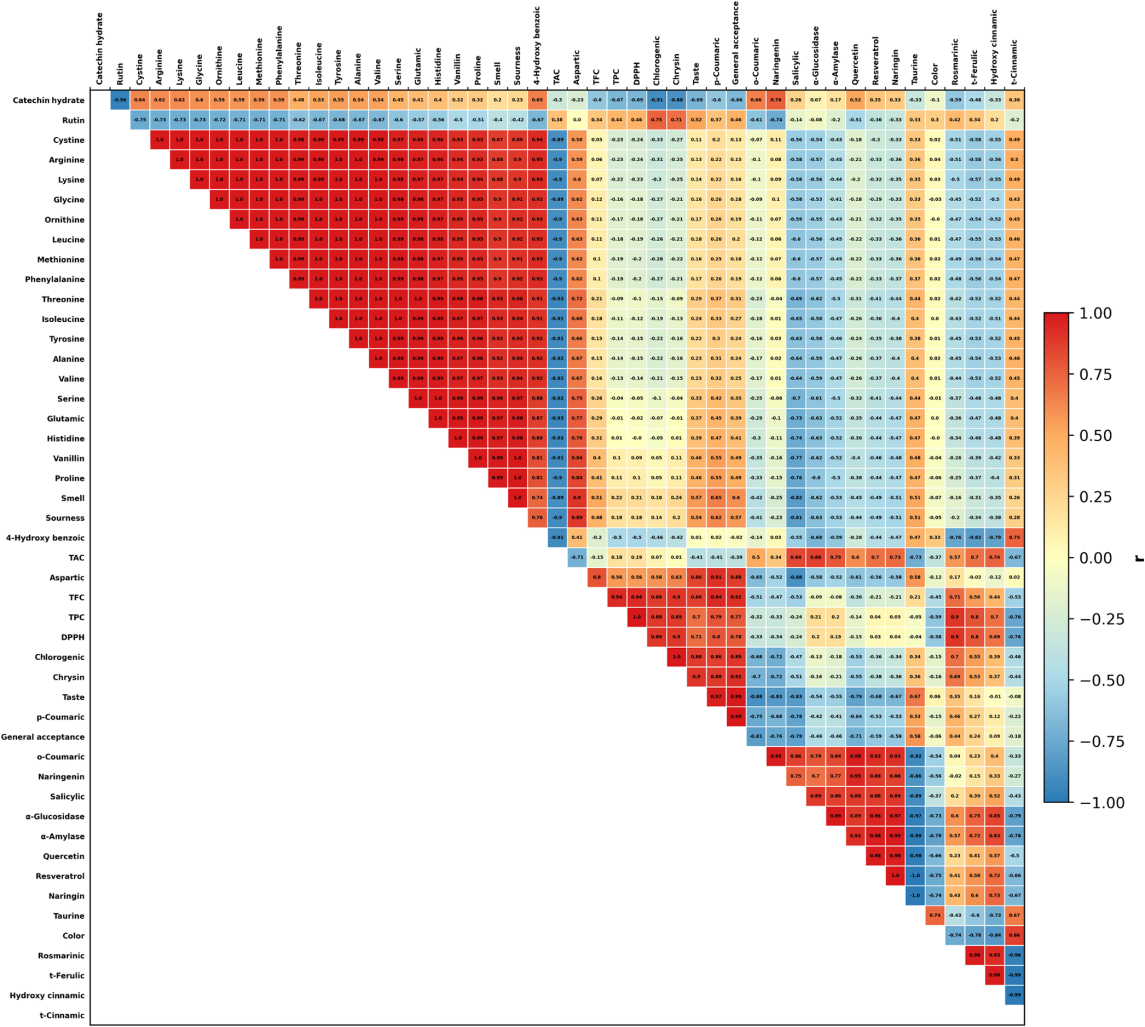
Pearson correlation heatmap (*r*) among phenolic compounds, amino acids, bioactive indices, enzyme inhibitory activities, and sensory attributes in blackthorn kombucha tea formulations. Cells display Pearson’s correlation coefficients (*r*). BKT: Blackthorn kombucha tea; PB-BKT: Propolis blackthorn kombucha tea; PL-BKT: Pollen blackthorn kombucha tea; BB-BKT: Bee bread blackthorn kombucha tea.

With respect to antioxidant capacity, the relationship between DPPH• radical scavenging activity and TPC was nearly linear (*r* = 0.9998). Moreover, strong positive correlations of DPPH• with TFC (*r* = 0.944) and rosmarinic acid (*r* = 0.902) indicate that phenolic enrichment is a primary driver of antioxidant performance. Among sensory subdimensions, the extremely high correlation between smell and sourness (*r* = 0.999), together with the synchronous variation of smell with proline (*r* = 0.991) and vanillin (*r* = 0.991), suggests that aroma perception may be jointly influenced by specific amino acids and aroma-related compounds. Nevertheless, given the limited sample size (*n* = 4), these correlations should be interpreted within an exploratory framework and validated using larger data sets. Despite this limitation, the strong concordance between PCA and Pearson correlation analyses consistently captures formulation-dependent differentiation.

The PCA biplot presented in Figure 6 reveals a distinct discrimination among the four kombucha formulations across the first two principal components. PC1 accounted for 56.10% of the overall variability, whereas the PC2 explained 26.91%, yielding a combined variance explanation of 83.00%. In terms of score distribution, the PL-BKT sample was distinguished by the highest positive loading along PC1 (PC1 = 8.35), whereas PB-BKT was positioned in the negative regions of both PC1 and PC2 (PC1 = −5.58; PC2 = −3.07). Conversely, BB-BKT exhibited a pronounced positive separation along PC2 (PC2 = 6.05), reflecting a distinct chemical and sensory profile.

**Figure 6:**
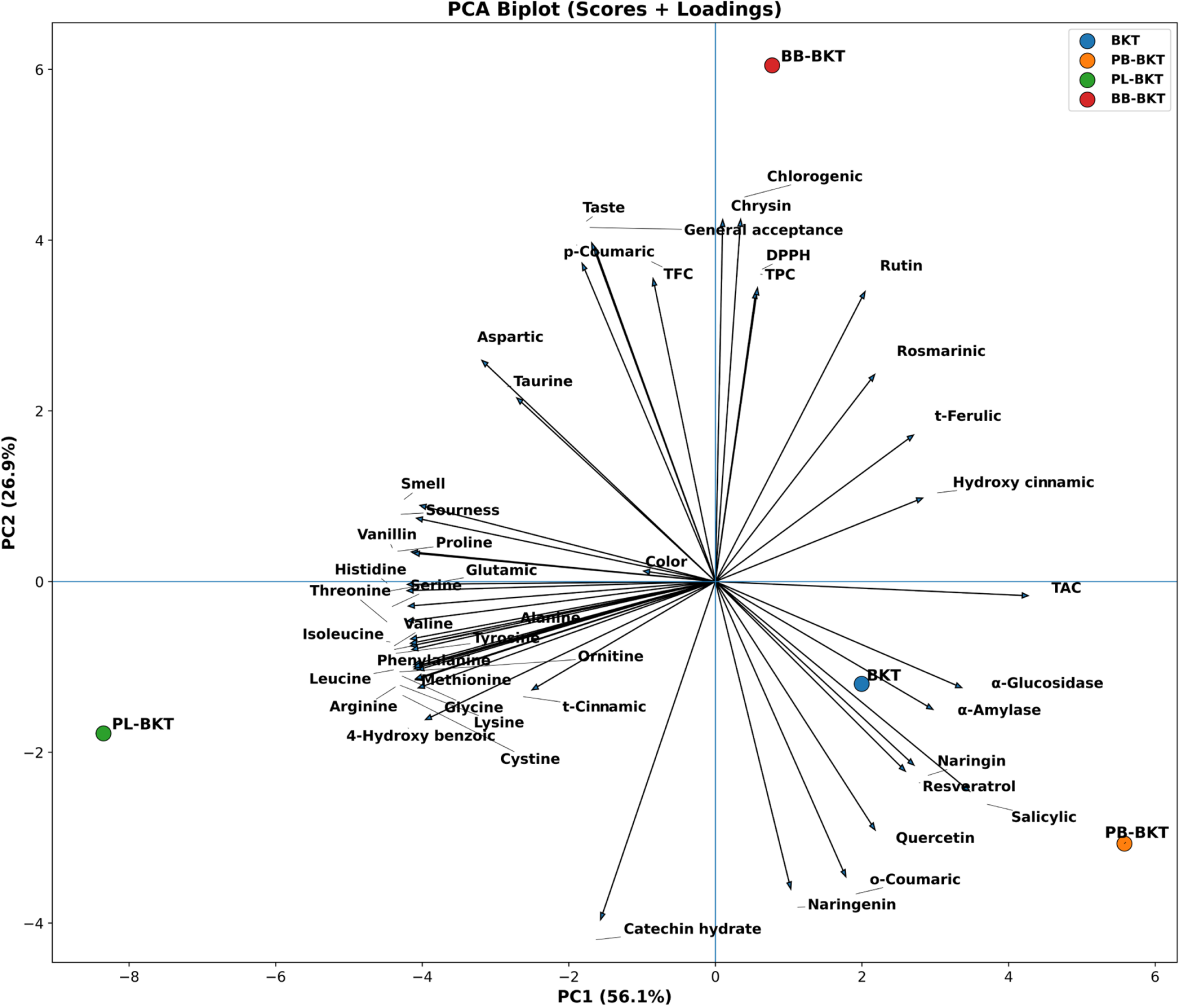
PCA biplot (scores and loadings) illustrating the multivariate separation of blackthorn kombucha tea formulations and the contribution of measured variables to PC1 and PC2. BKT: Blackthorn kombucha tea; PB-BKT: Propolis blackthorn kombucha tea; PL-BKT: Pollen blackthorn kombucha tea; BB-BKT: Bee bread blackthorn kombucha tea.

This multivariate separation is consistent with key quality indicators. BB-BKT exhibited the highest values of TPC (257.10), TFC (36.16), DPPH• activity (57.05), and sensory attributes (Taste = 8.25; General acceptance = 8.32), whereas PB-BKT was characterized by higher total acidity (TAC = 77.25) and stronger enzyme inhibitory activities against α-glucosidase (40.70) and α-amylase (42.02). Loading patterns further suggest that PC2 is primarily associated with specific phenolic compounds, notably chlorogenic acid (79.04) and chrysin (66.42), as well as sensory acceptance and taste attributes, whereas PC1 appears to be more strongly influenced by the amino acid profile. Overall, the formulation-based discrimination revealed by PCA, together with the robust association patterns identified by Pearson correlation analysis, demonstrate that supplementation with propolis, pollen, and bee bread markedly reshapes both the bioactive composition and the sensory characteristics of blackthorn kombucha.

## 4 Conclusions

This study demonstrated that kombucha enriched with *P. spinosa* L. juice and bee products (propolis, pollen, and bee bread) provides a robust model for functional beverage design through RSM-based optimization and advanced analytical characterization (HPLC–DAD, LC–MS/MS). From a sensory perspective, the highest overall acceptability was observed in the bee bread-enriched sample (8.32), which also exhibited the strongest antioxidant response (TPC 257.10 mg GAE/100 mL; TFC 36.16 mg CE/100 mL; DPPH• 57.05 μmol TE/mL). HPLC–DAD data revealed that total phenolics increased to 159.23–186.15 μg/mL following enrichment, and that marker compounds, such as chlorogenic acid and catechin derivatives, varied depending on the formulation. LC–MS/MS supported an increase in free amino acids (particularly proline) with pollen supplementation. Propolis addition highlighted the antidiabetic potential, yielding the highest enzyme inhibition (α-glucosidase: 40.7%; α-amylase: 42.02%). Although a partial reduction in bioactives was observed during in vitro digestion, the preservation of phenolic/flavonoid recovery within approximately the 21–30% range indicates that postdigestive activity may be sustained. Future studies are recommended to expand biomarkers using targeted/untargeted LC–HRMS-based metabolomic approaches, to correlate SCOBY microbiota (16S/ITS) with chemical profiles, and to evaluate shelf life and packaging stability (phenolic degradation kinetics, volatile profile), as well as in vivo/preclinical validation (glycemic response, bioaccessibility). A limitation of this work is that batch-specific botanical origin (palynological) identification of the commercial bee products was not performed; therefore, compositional variability due to botanical source cannot be fully excluded.

## Supplementary Material



## Notes

Supplementary tables: Table S1–S4, measured responses used in the RSM experimental design for blackthorn kombucha and enriched kombucha with propolis, pollen, and bee bread; Table S5–S8, ANOVA results of regression models for the combination tests (PDF)
