# Supplementary material for: From Chemistry to Functionality: HPLC–DAD/LC–MS/MS Characterization of Bee Product-Enriched Prunus spinosa L. Kombucha with In Vitro Antidiabetic Activity and Bioaccessibility
Source: ACS Omega. 2026 Feb 18;11(8):14048–63. doi: 10.1021/acsomega.6c00008 (PMC12961545; doi:10.1021/acsomega.6c00008)
Supplement: Supplementary file 1 [file ao6c00008_si_001.pdf]

From Chemistry to Functionality: HPLC–DAD/LC–MS/MS Characterization of Bee Product–Enriched *Prunus spinosa* L. Kombucha with *in Vitro* Antidiabetic Activity and Bioaccessibility

Melikenur Türkol[1], Çiğdem Yıldırım Maviş[1], Seydi Yıkmış[2]

[1]Nutrition and Dietetics, Faculty of Health Sciences, Halic University, 34060, Istanbul, Türkiye

[2]Department of Food Technology, Tekirdag Namık Kemal University, 59830, Tekirdag, Türkiye

Table S1. Measured responses used in the experimental design for RSM with blackthorn kombucha tea

Run no.	Independent variables		Dependent variables							
	A Blackthorn (% w/w)	B Black Tea (g/L)	Color		Taste		Smell		General Acceptance	
			Experimental data	RSM predicted	Experimental data	RSM predicted	Experimental data	RSM predicted	Experimental data	RSM predicted
**1**	12	8	7.63	7.64	7.33	7.34	6.91	6.88	7.01	6.97
**2**	12	11	7.23	7.26	6.95	6.97	7.12	7.12	7.13	7.16
**3**	14	9.5	7.72	7.67	7.42	7.36	7.08	7.08	7.05	7.10
**4**	10	12.5	7.47	7.44	7.18	7.13	6.57	6.56	6.58	6.57
**5**	12	11	7.23	7.26	6.95	6.97	7.12	7.12	7.13	7.16
**6**	12	14	8.32	8.33	7.92	7.95	6.83	6.86	6.87	6.91
**7**	12	11	7.23	7.26	6.95	6.97	7.12	7.12	7.18	7.16
**8**	12	11	7.23	7.26	6.95	6.97	7.12	7.12	7.18	7.16
**9**	14	12.5	7.72	7.67	7.42	7.33	7.40	7.34	7.41	7.39
**10**	16	11	7.57	7.6	7.23	7.27	7.21	7.23	7.22	7.23
**11**	12	11	7.32	7.26	6.95	6.97	7.12	7.12	7.18	7.16
**12**	10	9.5	6.78	6.76	6.52	6.5	6.80	6.84	6.86	6.92
**13**	8	11	6.46	6.46	6.21	6.21	6.23	6.21	6.24	6.23
**Optimization parameters**	14.47	14	8.17		7.79		7.35		7.43	
**Experimental values**			8.13± 0.06		8.05± 0.37		7.42± 0.1		7.35± 0.11	
**% Difference**			0.48%		3.34%		0.94%		1.07%	

A: Blackthorn (% w/w); B: Black Tea (g/L); RSM: Response surface methodology

Table S2. Measured responses used in the experimental design for RSM with blackthorn and propolis kombucha tea

Run no.	Independent variables			Dependent variables							
	A Blackthorn (% w/w)	B Black Tea (g/L)	C Propolis (% v/v)	Color		Taste		Smell		General Acceptance	
				Experimental data	RSM predicted	Experimental data	RSM predicted	Experimental data	RSM predicted	Experimental data	RSM predicted
**1**	12	11	1.5	7.58	7.58	7.35	7.35	6.93	6.97	8.05	8.07
**2**	16	14	1.5	7.15	7.18	6.94	6.95	6.47	6.51	7.01	7.10
**3**	16	8	1.5	7.19	7.2	6.97	6.98	6.61	6.63	7.13	7.04
**4**	12	14	2.0	7.84	7.86	7.60	7.62	7.22	7.22	7.82	7.73
**5**	12	11	1.5	7.58	7.58	7.35	7.35	6.98	6.97	8.12	8.07
**6**	12	14	1.0	7.82	7.77	7.53	7.51	7.16	7.14	7.84	7.76
**7**	12	8	2.0	7.35	7.39	7.13	7.15	6.79	6.81	7.08	7.17
**8**	8	11	1.0	6.58	6.64	6.56	6.58	6.21	6.25	6.35	6.34
**9**	16	11	2.0	6.90	6.84	6.77	6.75	6.39	6.35	6.71	6.72
**10**	8	14	1.5	7.05	7.03	6.83	6.83	6.52	6.50	6.86	6.95
**11**	16	11	1.0	7.68	7.69	7.45	7.46	7.08	7.06	7.43	7.44
**12**	12	11	1.5	7.58	7.58	7.35	7.35	6.98	6.97	8.02	8.07
**13**	8	11	2.0	7.16	7.15	6.95	6.94	6.59	6.61	6.75	6.75
**14**	12	8	1.0	7.85	7.82	7.61	7.6	7.23	7.24	7.34	7.44
**15**	8	8	1.5	6.63	6.6	6.43	6.42	6.11	6.07	6.21	6.13
**Optimization parameters**	13.17	11.45	1.02	7.85		7.62		7.23		7.96	
**Experimental values**				7.66± 0.13		7.23± 0.06		6.88± 0.08		7.51± 0.14	
**% Difference**				2.42%		5.11%		4.84%		5.65%	

A: Blackthorn (% w/w); B: Black Tea (g/L); C: Propolis (% v/v); RSM: Response surface methodology

Table S3. Measured responses used in the experimental design for RSM with blackthorn and pollen kombucha tea

Run no.	Independent variables			Dependent variables							
	A Blackthorn (% w/w)	B Black Tea (g/L)	C Pollen (% w/w)	Color		Taste		Smell		General Acceptance	
				Experimental data	RSM predicted	Experimental data	RSM predicted	Experimental data	RSM predicted	Experimental data	RSM predicted
**1**	12	11	5	7.28	7.26	6.91	6.88	6.65	6.71	7.65	7.64
**2**	16	14	5	6.81	6.84	6.52	6.53	6.27	6.28	6.66	6.74
**3**	16	8	5	6.90	6.90	6.55	6.56	6.35	6.38	6.70	6.64
**4**	12	14	7	7.53	7.53	7.15	7.17	6.94	6.94	7.27	7.21
**5**	12	11	5	7.28	7.26	6.91	6.88	6.71	6.71	7.65	7.64
**6**	12	14	3	7.51	7.47	7.08	7.06	6.73	6.76	7.56	7.47
**7**	12	8	7	7.06	7.09	6.70	6.72	6.52	6.49	6.73	6.81
**8**	8	11	3	6.37	6.41	6.17	6.19	5.96	5.97	6.07	6.09
**9**	16	11	7	6.58	6.54	6.37	6.34	6.13	6.13	6.37	6.35
**10**	8	14	5	6.77	6.77	6.42	6.41	6.26	6.23	6.52	6.57
**11**	16	11	3	7.38	7.38	7.01	7.01	6.80	6.77	7.14	7.14
**12**	12	11	5	7.22	7.26	6.83	6.88	6.78	6.71	7.62	7.64
**13**	8	11	7	6.87	6.87	6.53	6.52	6.33	6.36	6.41	6.41
**14**	12	8	3	7.53	7.53	7.19	7.17	6.94	6.94	6.97	7.03
**15**	8	8	5	6.37	6.33	6.05	6.04	5.87	5.86	5.90	5.82
**Optimization parameters**	13.41	11.15	3	7.57		7.19		6.94		7.61	
**Experimental values**				7.33± 0.16		7.75± 0.13		7.40± 0.08		7.32± 0.11	
**% Difference**				3.17%		7.22%		6.63%		3.81%	

A: Blackthorn (% w/w); B: Black Tea (g/L); C: Pollen (% w/w); RSM: Response surface methodology

Table S4. Measured responses used in the experimental design for RSM with blackthorn and bee bread kombucha tea

Run no.	Independent variables			Dependent variables							
	A Blackthorn (% w/w)	B Black Tea (g/L)	C Bea Bread (%)	Color		Taste		Smell		General Acceptance	
				Experimental data	RSM predicted	Experimental data	RSM predicted	Experimental data	RSM predicted	Experimental data	RSM predicted
**1**	12	11	5	6.97	6.97	7.57	7.57	7.18	7.18	7.45	7.45
**2**	16	14	5	6.58	6.59	7.09	7.10	6.23	6.30	6.57	6.61
**3**	16	8	5	6.65	6.64	7.18	7.20	6.37	6.34	6.76	6.79
**4**	12	14	8	7.21	7.22	7.83	7.84	7.41	7.33	7.35	7.37
**5**	12	11	5	6.97	6.97	7.57	7.57	7.18	7.18	7.45	7.45
**6**	12	14	2	7.19	7.16	7.64	7.64	6.87	6.85	7.32	7.29
**7**	12	8	8	6.76	6.79	7.34	7.34	6.54	6.55	6.92	6.95
**8**	8	11	2	6.12	6.14	6.76	6.77	5.98	5.97	6.35	6.41
**9**	16	11	8	6.29	6.27	6.97	6.96	6.15	6.16	6.54	6.48
**10**	8	14	5	6.49	6.50	7.03	7.01	6.27	6.29	6.67	6.64
**11**	16	11	2	7.07	7.08	7.68	7.66	6.82	6.76	7.24	7.23
**12**	12	11	5	6.97	6.97	7.57	7.57	7.18	7.18	7.45	7.45
**13**	8	11	8	6.59	6.57	7.15	7.17	6.35	6.40	6.74	6.75
**14**	12	8	2	7.22	7.22	7.84	7.84	7.12	7.20	7.46	7.44
**15**	8	8	5	6.10	6.09	6.62	6.61	5.88	5.81	6.25	6.21
**Optimization parameters**	11.31	13.75	8	7.22		7.84		7.34		7.40	
**Experimental values**				7.25± 0.04		7.72± 0.17		7.30± 0.06		7.33± 0.10	
**% Difference**				0.42%		1.53%		0.55%		0.95%	

A: Blackthorn (% w/w); B: Black Tea (g/L); C: Bee Bread (%); RSM: Response surface methodology

Table S5. ANOVA in the regression model of the combination test for blackthorn kombucha tea

		Color		Taste		Smell		General Acceptance	
**Source**	**DF**	**F-Value**	**P-Value**	**F-Value**	**P-Value**	**F-Value**	**P-Value**	**F-Value**	**P-Value**
**Model**	5	240.13	0.000	167.56	0.000	211.55	0.000	122.63	0.000
**Linear**	2	318.20	0.000	216.74	0.000	372.75	0.000	202.85	0.000
**A**	1	464.76	0.000	325.34	0.000	745.06	0.000	403.96	0.000
**B**	1	171.65	0.000	108.13	0.000	0.45	0.523	1.73	0.229
**Square**	2	253.89	0.000	181.14	0.000	120.90	0.000	76.15	0.000
**AA**	1	35.72	0.001	27.98	0.001	214.85	0.000	143.73	0.000
**BB**	1	366.17	0.000	256.95	0.000	83.52	0.000	38.6	0.000
**2-Way Interaction**	1	56.45	0.000	42.04	0.000	70.46	0.000	55.16	0.000
**AB**	1	56.45	0.000	42.04	0.000	70.46	0.000	55.16	0.000
**Error**	7								
**Lack-of-Fit**	3	1.54	0.334	*	*	*	*	4.73	0.084
**Pure Error**	4								
**Total**	12								
**R^2**		99.42		99.17		99.34		98.87	
**Adj. R^2**		99.01		98.58		98.87		98.07	
**Pred. R^2**		97.37		93.85		93.33		91.09	

A: Blackthorn; B: Black Tea; DF: degrees of freedom; R^2—coefficient of determination; $p < 0.05$, significant differences; $p < 0.01$, very significant differences; *The pure error was zero; thus, the Lack-of-Fit F-value and p-value could not be computed. This indicates that the model fits the data without detectable deviation

Table S6. ANOVA in the regression model of the combination test for blackthorn and propolis kombucha tea

		Color		Taste		Smell		General Acceptance	
**Source**	**DF**	**F-Value**	**P-Value**	**F-Value**	**P-Value**	**F-Value**	**P-Value**	**F-Value**	**P-Value**
**Model**	9	94.01	0.000	475.67	0.000	107.13	0.000	43.10	0.000
**Linear**	3	49.05	0.000	260.83	0.000	45.57	0.000	24.13	0.002
**A**	1	96.88	0.000	495.98	0.000	80.61	0.000	40.57	0.001
**B**	1	30.35	0.003	153.05	0.000	25.64	0.004	28.55	0.003
**C**	1	19.93	0.007	133.45	0.000	30.44	0.003	3.28	0.130
**Square**	3	165.9	0.000	872.83	0.000	202.83	0.000	93.59	0.000
**AA**	1	462.89	0.000	2383.45	0.000	546.12	0.000	261.36	0.000
**BB**	1	1.09	0.345	1.12	0.338	0.01	0.917	20.42	0.006
**CC**	1	14.08	0.013	130.98	0.000	35.92	0.002	19.32	0.007
**2-Way Interaction**	3	67.06	0.000	293.37	0.000	73.01	0.000	11.57	0.011
**AB**	1	18.12	0.008	100.33	0.000	38.83	0.002	10.80	0.022
**AC**	1	159.97	0.000	612.72	0.000	147.19	0.000	22.86	0.005
**BC**	1	23.09	0.005	167.05	0.000	33.01	0.002	1.05	0.353
**Error**	5								
**Lack-of-Fit**	3	*	*	*	*	2.55	0.294	8.02	0.113
**Pure Error**	2								
**Total**	14								
**R^2**		99.41%		99.88%		99.48%		98.73%	
**Adj. R^2**		98.35%		99.67%		98.56%		96.44%	
**Pred. R^2**		90.60%		98.13%		93.21%		80.98%	

A: Blackthorn; B: Black Tea; C: Propolis (% v/v); DF: degrees of freedom; R^2—coefficient of determination; $p < 0.05$, significant differences; $p < 0.01$, very significant differences; *The pure error was zero; thus, the Lack-of-Fit F-value and p-value could not be computed. This indicates that the model fits the data without detectable deviation

Table S7. ANOVA in the regression model of the combination test for blackthorn and pollen kombucha tea

		Color		Taste		Smell		General Acceptance	
**Source**	**DF**	**F-Value**	**P-Value**	**F-Value**	**P-Value**	**F-Value**	**P-Value**	**F-Value**	**P-Value**
**Model**	9	123.27	0.000	125.97	0.000	67.66	0.000	62.88	0.000
**Linear**	3	57.88	0.000	69.49	0.000	28.20	0.001	38.79	0.001
**A**	1	103.40	0.000	133.10	0.000	59.59	0.001	58.64	0.001
**B**	1	35.20	0.002	36.92	0.002	12.61	0.016	43.86	0.001
**C**	1	35.03	0.002	38.46	0.002	12.39	0.017	13.86	0.014
**Square**	3	221.50	0.000	229.16	0.000	123.11	0.000	133.19	0.000
**AA**	1	608.39	0.000	608.82	0.000	344.97	0.000	370.92	0.000
**BB**	1	1.62	0.260	0.08	0.788	0.88	0.391	35.04	0.002
**CC**	1	25.74	0.004	46.31	0.001	12.19	0.017	22.62	0.005
**2-Way Interaction**	3	90.43	0.000	79.26	0.000	51.66	0.000	16.66	0.005
**AB**	1	30.41	0.003	26.67	0.004	20.37	0.006	13.02	0.015
**AC**	1	210.10	0.000	160.85	0.000	97.81	0.000	36.91	0.002
**BC**	1	30.76	0.003	50.27	0.001	36.79	0.002	0.06	0.823
**Error**	5								
**Lack-of-Fit**	3	2.46	0.303	0.50	0.718	0.44	0.752	46.06	0.021
**Pure Error**	2								
**Total**	14								
**R^2**		99.55%		99.56%		99.19%		99.12%	
**Adj. R^2**		98.74%		98.77%		97.72%		97.55%	
**Pred. R^2**		94.14%		96.42%		93.74%		86.16%	

A: Blackthorn; B: Black Tea; C: Pollen (% w/w); DF: degrees of freedom; R^2—coefficient of determination; $p < 0.05$, significant differences; $p < 0.01$, very significant differences; *The pure error was zero; thus, the Lack-of-Fit F-value and p-value could not be computed. This indicates that the model fits the data without detectable deviation

Table S8. ANOVA in the regression model of the combination test for blackthorn and bee bread kombucha tea

		Color		Taste		Smell		General Acceptance	
**Source**	**DF**	**F-Value**	**P-Value**	**F-Value**	**P-Value**	**F-Value**	**P-Value**	**F-Value**	**P-Value**
**Model**	9	336.44	0.000	515.45	0.000	62.97	0.000	100.88	0.000
**Linear**	3	170.83	0.000	240.67	0.000	14.01	0.007	30.46	0.001
**A**	1	309.47	0.000	512.18	0.000	24.15	0.004	50.72	0.001
**B**	1	99.74	0.000	104.58	0.000	15.53	0.011	11.47	0.020
**C**	1	103.28	0.000	105.27	0.000	2.36	0.185	29.18	0.003
**Square**	3	591.93	0.000	941.10	0.000	139.66	0.000	219.06	0.000
**AA**	1	1653.50	0.000	2587.94	0.000	411.51	0.000	637.48	0.000
**BB**	1	4.84	0.079	9.31	0.028	16.65	0.010	36.35	0.002
**CC**	1	47.34	0.001	129.53	0.000	0.51	0.507	0.29	0.611
**2-Way Interaction**	3	246.56	0.000	364.59	0.000	35.25	0.001	53.14	0.000
**AB**	1	77.63	0.000	140.54	0.000	11.07	0.021	31.47	0.002
**AC**	1	576.18	0.000	684.02	0.000	43.38	0.001	101.02	0.000
**BC**	1	85.87	0.000	269.21	0.000	51.31	0.001	26.91	0.004
**Error**	5								
**Lack-of-Fit**	3	*	*	*	*	*	*	*	*
**Pure Error**	2								
**Total**	14								
**R^2**		99.84%		99.89%		99.13%		99.45%	
**Adj. R^2**		99.54%		99.70%		97.55%		98.47%	
**Pred. R^2**		97.36%		98.28%		86.01%		91.24%	

A: Blackthorn; B: Black Tea; C: Bee Bread (%); DF: degrees of freedom; R^2—coefficient of determination; $p < 0.05$, significant differences; $p < 0.01$, very significant differences; *The pure error was zero; thus, the Lack-of-Fit F-value and p-value could not be computed. This indicates that the model fits the data without detectable deviation
